# Structure of the transcribing RNA polymerase II–Elongin complex

**DOI:** 10.1038/s41594-023-01138-w

**Published:** 2023-11-06

**Authors:** Ying Chen, Goran Kokic, Christian Dienemann, Olexandr Dybkov, Henning Urlaub, Patrick Cramer

**Affiliations:** 1 Department of Molecular Biology, Max Planck Institute for Multidisciplinary Sciences, Göttingen, Germany; 2 Bioanalytical Mass Spectrometry, Max Planck Institute for Multidisciplinary Sciences, Göttingen, Germany; 3 University Medical Center Göttingen, Institute of Clinical Chemistry, Bioanalytics Group, Göttingen, Germany; 4 Cluster of Excellence ‘Multiscale Bioimaging: from Molecular Machines to Networks of Excitable Cells’ (MBExC), University of Göttingen, Göttingen, Germany; 5 Present Address: Department of Clinical Laboratory, Qilu Hospital of Shandong University, Jinan, China

**Keywords:** Cryoelectron microscopy, Transcription factors, Transcription

## Abstract

Elongin is a heterotrimeric elongation factor for RNA polymerase (Pol) II transcription that is conserved among metazoa. Here, we report three cryo-EM structures of human Elongin bound to transcribing Pol II. The structures show that Elongin subunit ELOA binds the RPB2 side of Pol II and anchors the ELOB–ELOC subunit heterodimer. ELOA contains a ‘latch’ that binds between the end of the Pol II bridge helix and funnel helices, thereby inducing a conformational change near the polymerase active center. The latch is required for the elongation-stimulatory activity of Elongin, but not for Pol II binding, indicating that Elongin functions by allosterically regulating the conformational mobility of the polymerase active center. Elongin binding to Pol II is incompatible with association of the super elongation complex, PAF1 complex and RTF1, which also contain an elongation-stimulatory latch element.

## Abstract (web summary)

Using cryo-EM, here the authors structurally delineate the Elongin–RNA polymerase II holocomplex. They show that Elongin allosterically regulates the transcribing RNA polymerase II via a latch that affects its conformational mobility.

## Main

Eukaryotic transcription by RNA polymerase II is regulated not only during initiation, but also during the elongation phase^1^. Elongin is an elongation factor that is thought to stimulate transcription by suppressing transient pausing of Pol II (refs. ^2–4^), especially at low ribonucleotide triphosphate concentrations^5–7^. Elongin was discovered as a heterotrimeric factor consisting of subunits Elongin A (ELOA), Elongin B (ELOB) and Elongin C (ELOC)^7–11^. ELOA alone can stimulate Pol II transcription elongation in vitro^8^. ELOA occurs as three different isoforms in humans, ELOA (ELOA1), ELOA2 (ref. ^12^) and ELOA3 (ref. ^13^). ELOB is a ubiquitin-like protein^14^. ELOC resembles the SCF ubiquitin ligase subunit Skp1 and forms the heterodimeric ELOB–ELOC subcomplex that enhances the activity of ELOA^8,14^. Trimeric Elongin can interact with the Cullin–RING E3 ubiquitin ligase CUL5–RBX2 complex, forming a pentameric complex^15,16^, which is involved in ubiquitylation and degradation of the RPB1 subunit of stalled Pol II upon ultraviolet irradiation^17–21^. Various stresses, including DNA damage, promote the assembly of the trimeric Elongin with CUL5–RBX2, converting Elongin from an elongation factor to an adaptor for Pol II ubiquitylation^22,23^. Here we refer to the trimeric complex as Elongin.

ELOA consists of a TFIIS N-terminal domain (TND)^24^, an unstructured middle region, a predicated Elongin A superfamily domain and an unstructured C-terminal region^25^. Studies of rat Elongin showed that ELOA interacts with the ELOB–ELOC subcomplex via a ten-residue BC-box motif on ELOA^26^ and interacts with Pol II via ELOA residues 590–690 (ref. ^25^). A C-terminal region of rat ELOA (residues 521–680, corresponding to human ELOA 548–707) is minimally required for the elongation-stimulatory activity^26^. Although Elongin was well characterized biochemically, the molecular mechanisms underlying Elongin interaction with Pol II and its elongation-stimulatory function remain elusive. Here we report the cryo-EM structures of Elongin in complex with Pol II and elongation factor SPT6 at a nominal resolution of 2.7–3.0 Å. The results show how Elongin binds to Pol II, reveal the exact regions required for the elongation-stimulatory function of Elongin and elucidate the mechanism of elongation stimulation by Elongin.

## Results

### Stimulation of Pol II elongation by recombinant human Elongin

**Fig. 1:**
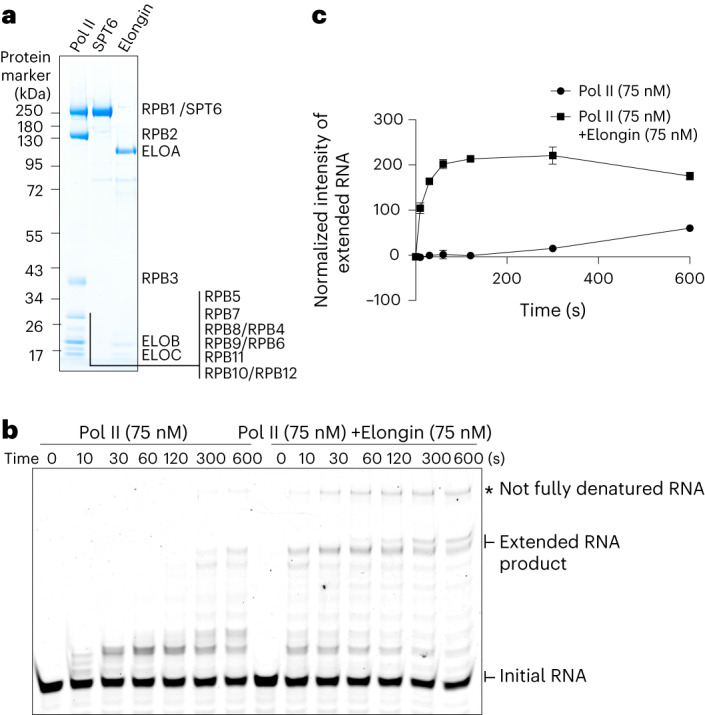
Elongin stimulates Pol II transcription elongation. **a**, Preparation of Pol II, SPT6 and Elongin. About 7.2 picomoles of each complex was analyzed by SDS–PAGE followed by Coomassie blue staining. The SDS–PAGE was performed once, showing the proteins from one of the purification batches. (See unprocessed gel images in the source data for this figure.) **b**, RNA extension assays in the absence or presence of Elongin at 30 °C over a time span of 0–600 s. The gel images show the fluorescence signals of 5′-FAM label on initial RNA and extended RNA products, as indicated. Fully extended RNA products are 51 nucleotides in length. (See unprocessed gel images in the source data for this figure.) **c**, Quantification of extended RNA products in **b**. Means and standard deviations of the normalized intensity of RNA product from three independent experiments are shown as dots and error bars against time (*n* = 3). (See statistical source data for this figure.) Source data

**Extended Data Fig. 1:**
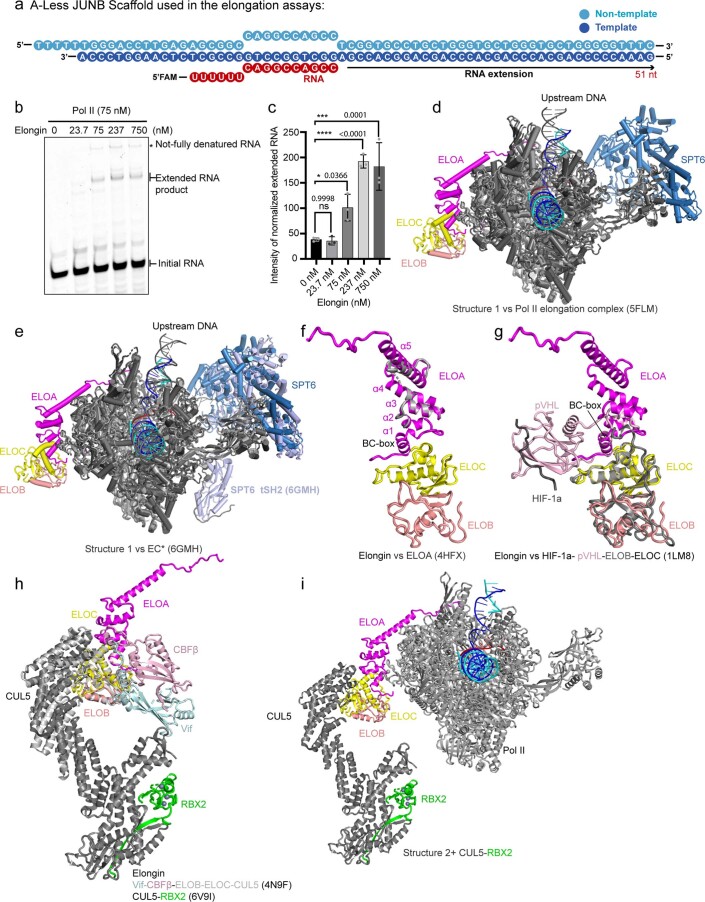
Testing of Elongin activity by RNA extension assays and comparison of structures. **a**. Scheme of the A-Less JUNB DNA-RNA hybrid scaffold for RNA extension assays. **b**. Titration of Elongin in RNA extension assays. **c**. Quantification of the extended RNA products in **b**. Means and standard deviations from three independent experiments were plotted as bar diagram and error bars (n = 3). The amount of extended RNA in the presence of Elongin were compared to the control reaction in the absence of Elongin. The P values are calculated by ordinary one-way ANOVA in GraphPad Prism 9 and labeled in the panel. ns, *, **, *** and **** indicates P > 0.05, P ≤ 0.05, P ≤ 0.01, P ≤ 0.001 and P ≤ 0.0001, respectively. Individual data points are shown as grey dots. See unprocessed gels and statistical source data in Source Data Extended Data Fig. 1. **d,e**. Superposition of Pol II-SPT6-Elongin structure with ELOA latch (structure 1) and Pol II elongation complexes by aligning the active site domain. **d**. structure 1 vs Pol II elongation complex (PDB: 5FLM)^28^. **e**. Structure 1 vs Pol II-SPT6-PAF complex (EC*, PDB: 6GMH^29^). Pol II in structure 1 is colored in light grey. Pol II elongation complex and EC* are colored in dark grey, except from SPT6 (light blue). In **e**, PAF is omitted for clarity. **f**. Superposition of Elongin and the ELOA superfamily homology domain (PDB: 4HFX). ELOA in 4HFX is colored in light grey. **g**. Superposition of Elongin and HIF-1a-pVHL-ELOC-ELOB (PDB: 1LM8^34^). HIF-1a, pVHL, ELOC and ELOB in 1LM8 are colored in deep grey, pink, light grey and medium grey, respectively. **h**. Superposition of Elongin, Vif-CBFβ-CUL5-ELOB-ELOC and CUL5-RBX2 complexes. The Vif-CBFβ-CUL5-ELOB-ELOC complex (PDB: 4N9F)^32^ was superposed to the ELOB-ELOC subcomplex of Elongin, with RMSD of 1.899 over158 CA atoms. The CUL5-RBX2 complex (PDB: 6V9I)^31^ was superposed to CUL5 residues 1–116 of 4N9F, with RMSD of 1.439 over 116 CA residues. CUL5 1-116 interacts with ELOC-ELOB and the following CUL5 domain has different curvatures in 4N9F and 6V9I. **i**. A model of the Pol II-ELOA-ELOB-ELOC-CUL5-RBX2 complex based on the superposition in **h**. Source data

We prepared human Elongin (Fig. 1a) and analyzed its transcription elongation activity using RNA extension assays in vitro (Methods, Fig. 1b,c and Extended Data Fig. 1a–c). We assembled a Pol II elongation complex on a DNA–RNA scaffold as described^27^ (Extended Data Fig. 1a), and incubated it with increasing amounts of recombinant Elongin. We started transcription by addition of CTP, UTP and GTP at final concentrations of 10 µM. A clear stimulation of RNA synthesis was observed when Elongin was added in a 1:1 molar ratio to Pol II (Extended Data Fig. 1b,c). Time-course experiments further showed that in the presence of Elongin the amount of extended RNA after 10–30 s was comparable to the products generated by Pol II alone after 5–10 min (Fig. 1b,c). This indicated that our recombinant human Elongin strongly stimulated Pol II transcription elongation, consistent with previous findings^8^. These results also showed that our recombinant Elongin complex was functionally active and could be used for structural analysis.

### Four structures obtained by cryo-EM analysis

**Extended Data Fig. 2:**
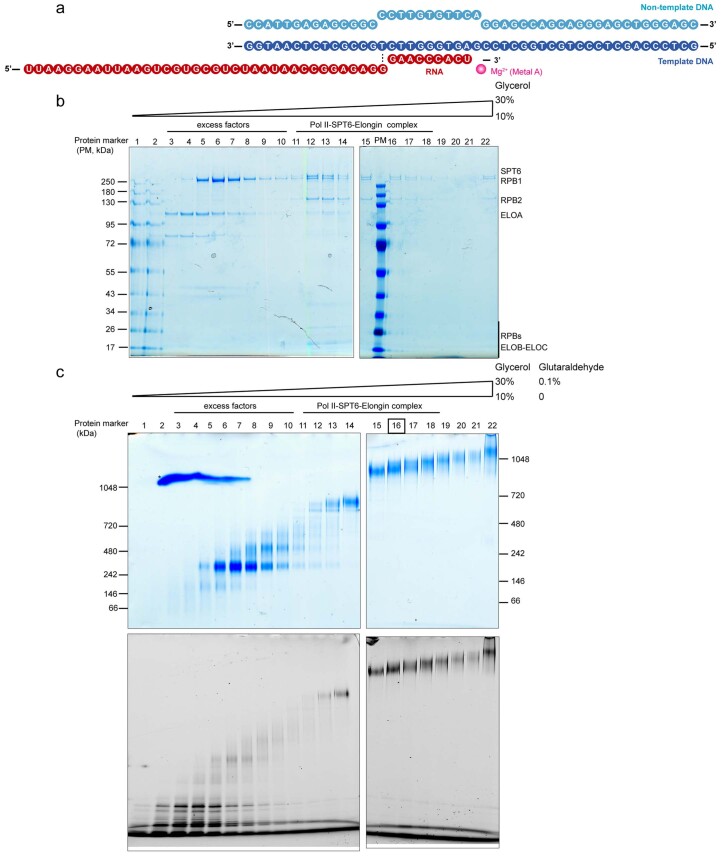
Preparation of the Pol II-SPT6-Elongin complex for cryo-EM analysis. **a**. Scheme of the DNA-RNA scaffold used in complex assembly. The 11-base mismatch in the DNA scaffold mimics the transcription bubble. The designed DNA-RNA hybrid contains 9 base pairs. RNA, template DNA and non-template DNA are colored in red, blue and cyan, respectively. The circle in magenta represents the position of Mg^2+^ metal A. The dash line indicates the (10^th^) T-G mismatch DNA-RNA base pair observed in the cryo-EM structure. **b** and **c**. Assembly of the Pol II-SPT6-Elongin complex by density gradient ultracentrifugation in native glycerol gradient (**b**) or in the presence of cross-linker Glutaraldehyde (**c**). In panel **b**, the gels show the distribution of 30 picomoles protein complexes in the glycerol gradient after fractioning into 200 µl fractions from the top to bottom of the gradient. 15 µl of each fraction were analyzed on SDS-PAGE, followed by Coomassie stain. In panel **c**, the gels show Native PAGE analysis of GraFix sample containing 90 picomoles of complexes followed by protein complex detection with Coomassie stain (upper panels) or by scanning the fluorescence signals of the 5’-FAM label RNA^56^. Fraction 16 of the GraFix sample was used for high-resolution data collection. * indicates accidental contamination by Coomassie precipitation. Details on complex formation are described in Methods. The complex assembly in b and c was performed once, respectively. More replication was not needed, because these two assemblies were enough to acquire high quality Cryo-EM data. See unprocessed gels in Source Data Extended Data Fig. 2. Source data

To study the structural basis for the elongation-stimulatory activity of Elongin, we used single-particle analysis cryo-EM. For initial analysis, we tried binding Elongin to various Pol II elongation complexes containing elongation factors DSIF and SPT6, among which the Pol II–SPT6–Elongin complex showed highest occupancy for Elongin in the cryo-EM experiments. We subsequently focused on the Pol II–SPT6–Elongin complex for high-resolution cryo-EM analysis. The complex was assembled on a nucleic acid scaffold containing a nine-base pair DNA–RNA hybrid within an eleven-nucleotide mismatch region that mimics a natural DNA bubble and was subjected to cryo-EM analysis (Extended Data Fig. 2a–c and Methods).

**Extended Data Fig. 3:**
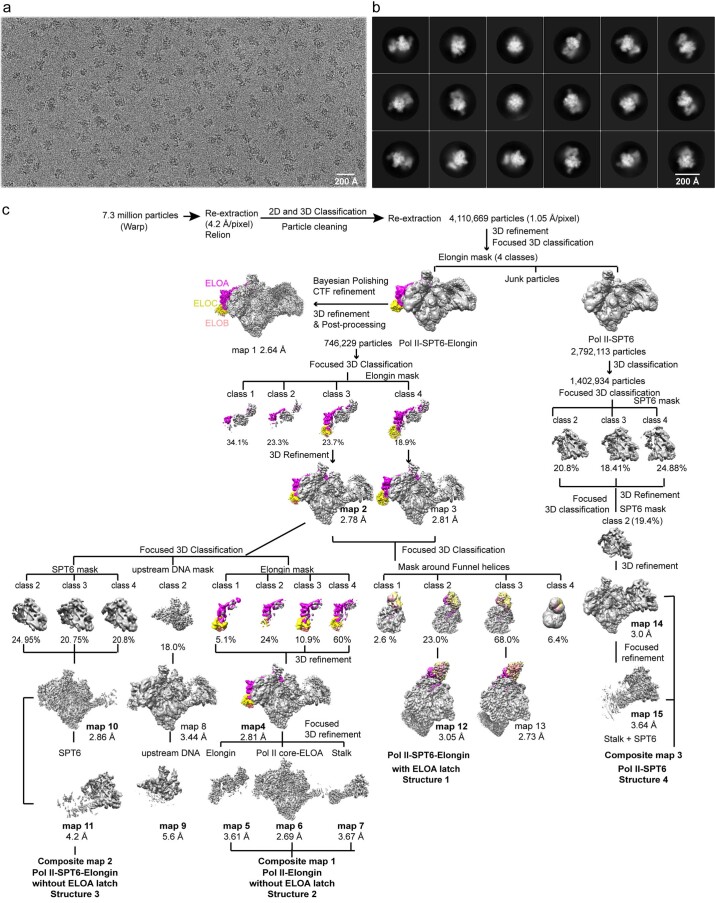
Cryo-EM data collection and processing. **a**. Representative micrograph. White scale bar indicates 200 Å. The micrograph represents one of the 35,579 micrographs that were collected from one Cryo-EM grid. **b**. Representative 2D classes. White scale bar indicates 200 Å. **c**. Scheme of data processing procedure.

**Extended Data Fig. 4:**
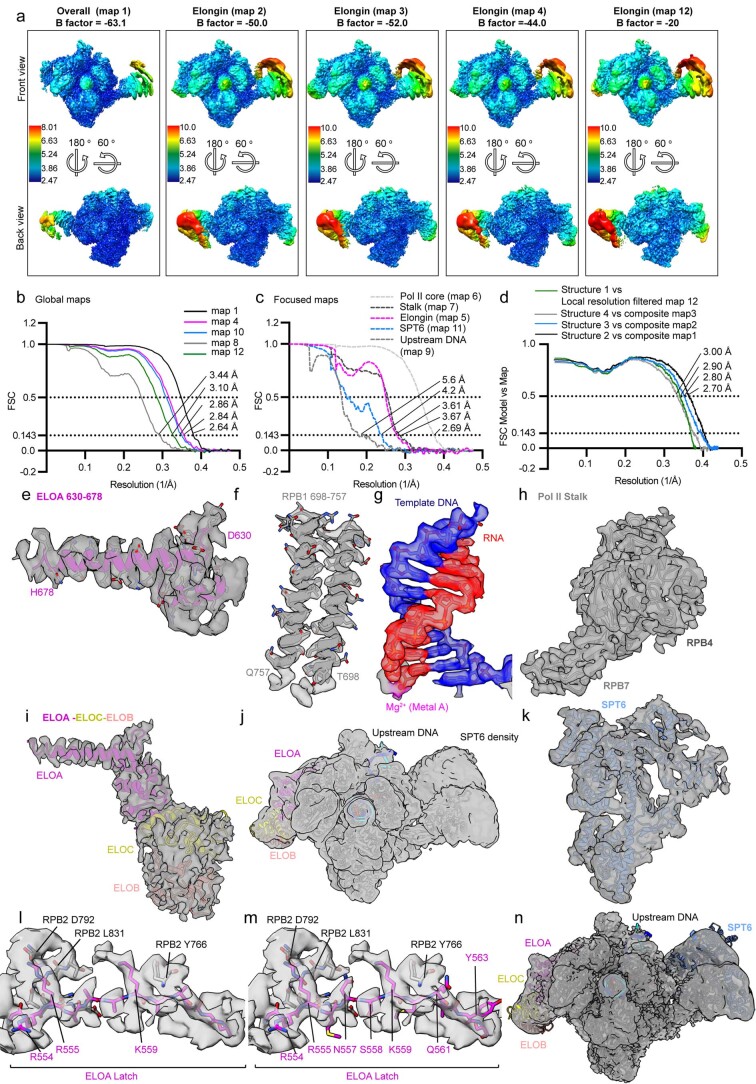
Quality of EM maps. **a**. Local resolution estimation of global maps. The local resolution filtered maps are colored with local resolution value estimated in Relion at indicated B factors. **b**. Fourier shell curves (FSC) for global maps, including map 1, map 4, map 10, map 8 and map 12. The resolution of maps is determined based on gold standard FSC = 0.143. **c**. Fourier shell curves for focused maps, including focused map for Pol II core (map 6), stalk (map 7), Elongin (map 5), SPT6 (map 11) and upstream DNA (map 9). **d**. Fourier shell curves indicate the ‘model to map’ fit for Pol II-Elongin lacking ELOA latch (structure 2), Pol II-SPT6-Elongin lacking ELOA latch (structure 3), Pol II-SPT6 (structure 4) and Pol II-SPT6-Elongin with ELOA latch (structure 1) models. The resolution of models is determined based on gold standard FSC = 0.5. **e-i**. Representative maps to show the fitting of structure 2 to composite map 1. In **e-g**, ELOA, RPB1 funnel helices and DNA-RNA hybrid are shown as cartoon presentation and the sidechains are shown as sticks. Mg^2+^ is shown as a magenta sphere. In **h-i**, Pol II stalk, Elongin are shown as cartoon presentation. Maps are shown as semi-transparent grey surface. **j**. Fitting of structure 2 into the consensus map 4, low-passed filtered to 6 Å. **k**. Fitting of SPT6 from structure 3 into composite map 2. **l**. Fitting of ELOA latch in structure 1 into local resolution filtered map 12. **m**. Fitting of ELOA latch in structure 1 into local resolution filtered map 12. The side chains of the ELOA latch residues other than G553, R554, R555 and K559 are placed in one of the most probable rotamers to indicate their position. In the deposited model for structure 1, these side chains are truncated as stubs as shown in panel l. **n**. Fitting of structure 1 into local resolution filtered global map 12.

We collected 35,579 micrographs that contained ~7.3 million single particles and performed extensive data processing (Extended Data Fig. 3a–c). Single-particle classification and refinements resulted in an overall reconstruction of the Pol II–SPT6–Elongin complex at a nominal resolution of 2.6 Å (Extended Data Fig. 3c, map 1). However, due to incomplete occupancy and flexibility of protein factors at the periphery, the local resolutions for the Pol II stalk, SPT6, upstream DNA and Elongin were lower (Extended Data Figs. 3c and 4a). Further classification and refinement with local masks resulted in focused maps for the Pol II stalk, SPT6, upstream DNA and Elongin at 3.7 Å, 4.2 Å, 5.6 Å and 3.6 Å, respectively (Extended Data Figs. 3c and 4b,c). Additionally, we obtained a detailed map for an ELOA N-terminal linker (residues 553–564), called here the ‘latch’, with the use of focused three-dimensional (3D) classification and global refinement (Extended Data Figs. 3c and 4l,m).

**Extended Data Fig. 5:**
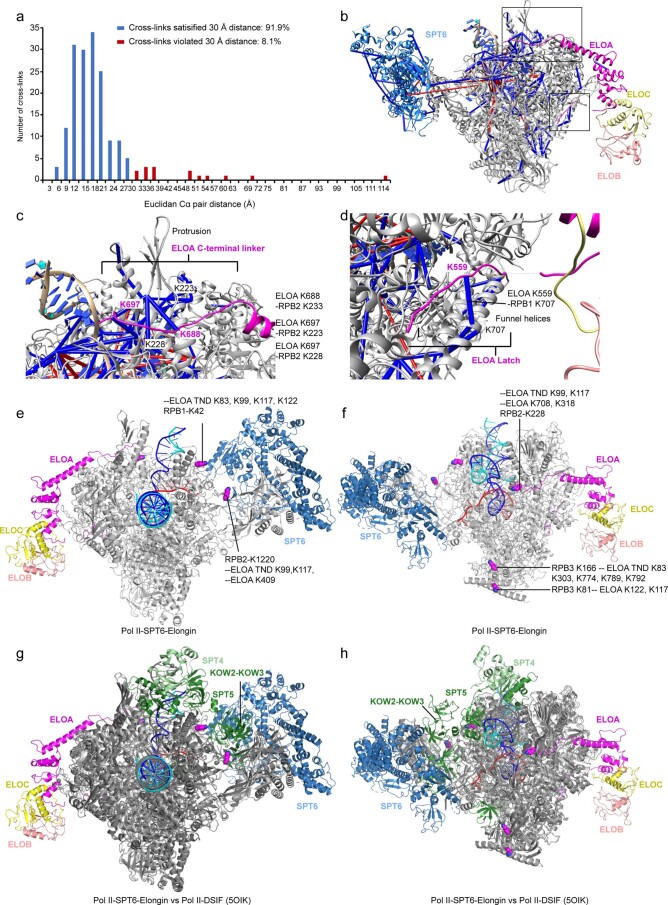
Mapping of cross-links onto Pol II-SPT6-Elongin model. **a**. Frequency distribution of the Cα-Cα distances between BS3-cross-linked residues. The number of unique cross-links is plotted against the Euclidean distance between Cα atoms of the two cross-linked residues. The histogram shows number of cross-links that falls into a distance range bin (3-6 Å, 6–9 Å, ect.). 30 Å is the maximum distance possible between Cα atoms of BS3-cross-linked Lys pairs in fully stretched conformation. In blue are the bins that satisfy a distance criterium of <30 Å, in red are those that violate it. **b**. Mapping of cross-links onto the Pol II-SPT6-Elongin structure (structure 1), corresponding to the plot in panel a. Cross-links are shown as solid lines and color coded is in **a**. **c**. Close-up view of the cross-links between ELOA C-terminal linker (magenta) and Pol II protrusion (grey). There are 91 cross-linked peptide spectrum matches (CSMs) of the ELOA K688-RPB2 K223 cross-link, 15 CSMs of the ELOA K697-RPB2 K223 cross-link and 41 CSMs of the ELOA K697-RPB2 K228 cross-link. **d**. Close-up view of the cross-link between ELOA latch (magenta) and Pol II funnel helices (grey). There are 4 CSMs of the ELOA K559-RPB1 K707 cross-link. **e, f**. Cross-linking sites of ELOA TND on Pol II surface, front view (e) and back view (f). The Pol II lysine residues that cross-link to ELOA TND are shown as spheres. **g, h**. Superposition of the Pol II-DSIF structure (PDB: 5OIK^52^) onto the Pol II-SPT6-Elongin structure, front view (g) and back view (h). The Pol II lysine residues that cross-link to ELOA TND are shown as spheres. See cross-links in Supplementary Data 1.

We obtained four structures of the transcribing Pol II elongation complexes (Extended Data Figs. 3c and 4a–n and Table 1). Structure 1 was built into a globally refined map (map 12) and represents the complete complex structure, containing Pol II, SPT6 and Elongin, including ELOB, ELOC and ELOA C-terminal region (residues 571–698), and the ELOA latch (residues 553–564) (Extended Data Figs. 3c and 4d,l,m). Structure 2 was obtained by building into composite map 1 and contains Pol II and Elongin and lacks the ELOA latch (Extended Data Fig. 4d–j). Structure 3 was built into composite map 2 and contains Pol II, SPT6 and Elongin and also lacks the ELOA latch (Extended Data Figs. 3c and 4k). Structure 4 was built into composite map 3 and contains Pol II and SPT6 (Extended Data Figs. 3c and 4d). The structures are supported by reliable cross-linking mass spectrometry data, which revealed robust cross-links at ELOA–Pol II interfaces observed in the structures (Extended Data Fig. 5a–d and Supplementary Data 1).

**Table 1:** Cryo-EM data collection, refinement and validation statistics

	Map 5 Elongin focused (EMD-16830)	Map 6 Pol II core focused (EMD-16831)	Map 7 stalk focused (EMD-16832)	Map 10 SPT6 global (EMD-16833)	Map 11 SPT6 focused (EMD-16834)	Map12 Elongin global (EMD -16836)	Map 14 Pol II–SPT6 global (EMD-16828)	Map 15 Pol II–SPT6 focused (EMD-16829)
**Data collection and processing**								
Magnification	×81,000	×81,000	×81,000	×81,000	×81,000	×81,000	×81,000	×81,000
Voltage (kV)	300	300	300	300	300	300	300	300
Electron exposure (e^–^/Å^2^)	40.09	40.09	40.09	40.09	40.09	40.09	40.09	40.09
Defocus range (μm)	0.35–7.50	0.35–7.50	0.35–7.50	0.35–7.50	0.35–7.50	0.35–7.50	0.35–7.50	0.35–7.50
Pixel size (Å)	1.05	1.05	1.05	1.05	1.05	1.05	1.05	1.05
Symmetry imposed	*C*1	*C*1	*C*1	*C*1	*C*1	*C*1	*C*1	*C*1
Initial particle images (no.)	177,945	177,945	177,945	177,945	177,945	318,472	896,636	896,636
Final particle images (no.)	136,189	136,189	136,189	118,642	118,642	72,087	174,029	174,029
Map resolution (Å)	3.61	2.69	3.67	2.86	4.2	3.05	3.04	3.64
FSC threshold	0.143	0.143	0.143	0.143	0.143	0.143	0.143	0.143
Map resolution range (Å)	N/A	N/A	N/A	2.61–13.2	N/A		2.71–15.0	N/A
Map sharpening *B* factor (Å^2^)	−124.0	−48.4	−103.3	−55.9	−123.7	−49.2	−103.3	−168.5
**Refinement map**	Composite map 1 (EMD-16838)			Composite map 2 (EMD-16837)		Local resolution filtered map 12 (EMD-16840)	Composite map 3 (EMD-16835)	
Current model	Structure 2 (PDB 8OEW)			Structure 3 (PDB 8OEV)		Structure 1 (PDB 8OF0)	Structure 4 (PDB 8OEU)	
Initial model used (PDB code)	7OKX (Pol II), AlphaFold2 (ELOA–ELOB–ELOC complex)			Structure 2 (this study), 7OOP (SPT6)		Structure 2 (this study)	Structure 2 (this study), 7OOP (SPT6)	
Model resolution (Å)	2.80			2.80		3.0	3.00	
FSC threshold	0.50			0.50		0.5	0.50	
Model–map correlation coefficients CC (masked)	0.82			0.77		0.82	0.77	
Model composition								
Non-hydrogen atoms	35,037			41,781		41,700	39,225	
Protein residues	4,180			5,005		5,010	4,683	
Nucleotides	77			77		75	79	
Ligands	9			9		9	9	
*B* factors (Å^2^)								
Protein	39.38			49.03		71.22	65.51	
Nucleotides	131.58			131.58		176.27	212.48	
Ligand	83.18			83.18		110.64	102.34	
R.m.s. deviations								
Bond lengths (Å)	0.005			0.005		0.006	0.006	
Bond angles (°)	0.775			1.101		1.124	0.876	
**Validation**								
MolProbity score	1.54			1.70		1.69	1.57	
Clashscore	6.09			8.88		8.05	6.60	
Poor rotamers (%)	0			0		0.02	0	
Ramachandran plot								
Favored (%)	96.68			96.55		96.31	96.75	
Allowed (%)	3.32			3.45		3.69	3.25	
Disallowed (%)	0			0		0	0	

### Structure of transcribing Pol II–SPT6–Elongin complex

**Fig. 2:**
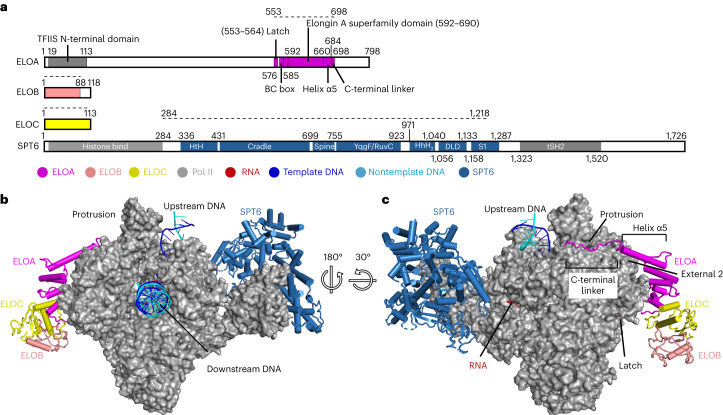
Structure of transcribing Pol II–SPT6–Elongin complex. **a**, Scheme for the domain organization of ELOA, ELOB, ELOC and SPT6 proteins. The modeled regions are indicated by the dashed line and colored as for the corresponding proteins. The color scheme is shown at the bottom by colored circles. **b**,**c**, Overview of the Pol II–SPT6–Elongin structure (structure 1): front view (**b**) and back view (**c**). Pol II is shown as surface presentation. ELOA, ELOB, ELOC and SPT6 are shown as cartoon presentation. Pol II, ELOA, ELOB, ELOC, SPT6, template DNA, nontemplate DNA and RNA are colored in gray, magenta, salmon, yellow, sky blue, blue, cyan and red, respectively.

The most complete Pol II–SPT6–Elongin model (structure 1) consists of Pol II, SPT6, ELOA C-terminal region (residues 553–698), including the latch, the BC box, the Elongin A superfamily domain and the C-terminal linker, ELOB and ELOC (Fig. 2a–c). The overall structure of the Pol II–SPT6–Elongin complex resembles the structure of the mammalian Pol II elongation complex^28^ (PDB 5FLM) (Extended Data Fig. 1d), but additionally contains SPT6 and Elongin, which bind to opposite sides of Pol II (Fig. 2b,c). SPT6 binds to the Pol II stalk, as observed in the active elongation complex EC*^29^, but lacks the SH2 domain and adopts several orientations, indicating flexibility (Fig. 2a and Extended Data Figs. 1e and 3c). Elongin binds on the other side, to the Pol II RPB2 domains ‘external 2’ and ‘protrusion’. Additionally, the ELOA latch binds to Pol II near the bridge helix and the funnel helices of the largest Pol II subunit RPB1. The upstream DNA shows a similar orientation as in the EC* complex (Extended Data Fig. 1e). Although not visible in the density, our cross-linking data indicate that the ELOA N-terminal region resides near the Pol II-binding site of the DSIF subunit SPT5 KOW2–KOW3 domain (Extended Data Fig. 5e–h). This suggested that DSIF may interfere with ELOA binding to Pol II.

### Elongin structure

**Fig. 3:**
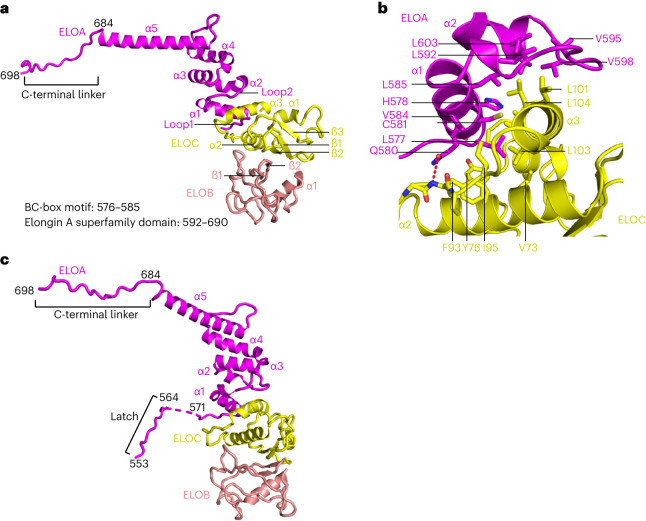
Structure of the Elongin complex. **a**, Overview of the Elongin complex. ELOA, ELOB and ELOC (structure 2) are shown as cartoon representation. **b**, Close-up view of the ELOA–ELOC interaction interface. Residues on the interface are shown as sticks. Hydrogen bonds are shown as dashed line. **c**, Overview of the Elongin complex. ELOA, ELOB and ELOC (structure 1) are shown as cartoon representation. The ELOA C-terminal linker and the latch are indicated.

Although Elongin was identified decades ago and many structures comprising the ELOB–ELOC subcomplex and its partners have been reported^30–32^, the structure of the trimeric Elongin has been lacking. We can now describe the Elongin structure as part of the larger elongation complex structure. The structure shows that ELOA, ELOB and ELOC form a heterotrimeric complex, as previously reported^26^. ELOA comprises five α-helices and four loops (Fig. 3a–c). Whereas Loop1–α1 forms the BC-box motif and interacts with ELOC, Loop2–α5 and a C-terminal extension form the predicted ELOA superfamily homology domain (Figs. 2a and 3a). The fold of ELOA α2–α4 and half of α5 is similar to the crystal structure of the ELOA superfamily homology domain, with a root mean squared deviation (r.m.s.d.) of 0.975 over 40 Cα atoms (PDB 4HFX chain A; Extended Data Fig. 1f). However, in our structure, α5 is longer and more straight compared to the previous crystal, which is probably due to the presence of Pol II (Extended Data Fig. 1f).

**Extended Data Fig. 6:**
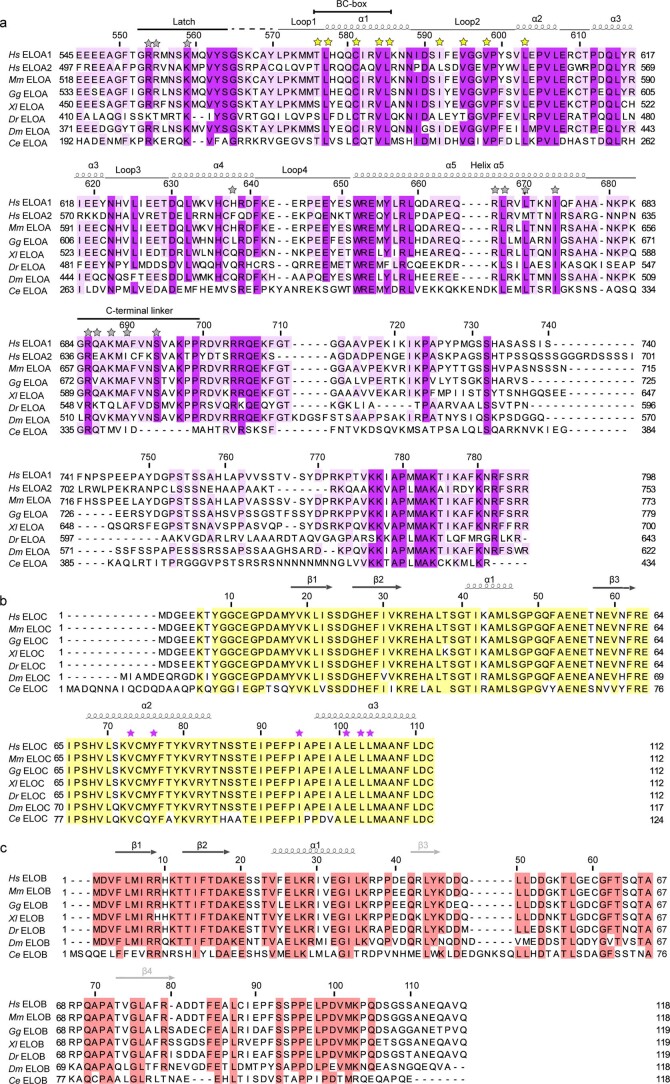
Sequence alignments of ELOA, ELOC and ELOB. **a**. Alignment of ELOA proteins from different species, including human (Homo sapiens *Hs* ELOA1 and ELOA2, UniProt AC: Q14241, Q8IYF1), mouse (*Mus musculus Mm* ELOA1, UniProt AC*:* Q8CB77), chicken (*Gallus gallus Gg* ELOA, UniProt AC: A0A1D5PAE7), frog (*Xenopus laevis Xl* ELOA, UniProt AC: A0A1L8HFS2), zebra fish (*Danio rerio Dr* ELOA, UniProt AC: Q803L4), fruit fly (*Drosophila melanogaster Dm* ELOA, UniProt AC: Q9VCP0) and worm (*Caenorhabditis elegans Ce* ELOA, UniProt AC*:* Q09413*)*. Yellow and grey stars indicate ELOA residues locating on the ELOA-ELOC interface and ELOA-Pol II interface, respectively. Residues with > 60% identity among species are highlighted with light and deep purple background. **b**. Alignment of ELOC proteins from different species, including human (*Hs*), mouse (Mm), chicken (*Gg*), frog (*Xl*), fruit fly (*Dm*), fish (*Dr*) and worm (*Ce)* with Uniprot AC or GenBank ID of Q15369, P83940, NP_001007889.1, XP_018098053.2, Q7JWD6, NP_001002440.2 and Q9BKS1, respectively. The species in ELOB and ELOC alignments are less than the ones in ELOA alignments, because ELOB and ELOC of some species are not present in the database. Residues with > 75% identity are highlighted with yellow background. Purple stars indicate ELOC residues locating on the ELOA-ELOC interface. **c**. Alignment of ELOB proteins from different species, including human (*Hs*), mouse (Mm), chicken (*Gg*), frog (*Xl*), fruit fly (*Dm*), fish (*Dr*) and worm (*Ce)* with UniProt AC or GenBank ID of Q15370, P62869, XP_040512765.1, NP_001080414.1, O44226, NP_001136426.1 and G5ECR7, respectively. Residues with > 75% identity are highlighted with salmon background. Secondary structure assignment is shown according to the structure of human ELOA, ELOB and ELOC from this study. Secondary structures were assigned by PyMOL dssp. Note: ELOB β3 and β4 were shown to be beta strands in other studies but not assigned by Pymol dssp in this study.

ELOC consists of three β-strands and three α-helices (Fig. 3a). Helix α3 mainly contributes to the interaction between ELOC and the BC box of ELOA, forming an interface of about 960 Å^2^ as calculated by PISA^33^ (Fig. 3b and Extended Data Fig. 6a,b). The interaction is mainly hydrophobic and is facilitated by hydrogen bonds (Fig. 3b). The ELOA–ELOC interaction interface is highly similar to the pVHL–ELOC interaction interface reported in the HIF-1α–pVHL–ELOC–ELOB structure (PDB 1LM8)^34^, as shown by the superposition of these structures (Extended Data Fig. 1g). This is also consistent with the prediction by sequence conservation^8^.

ELOB interacts with ELOC as reported in previous structures in the absence of Pol II, forming an interaction interface of ~910 Å^2^ according to PISA^33^. The interface is mediated by main chain hydrogen bonds and hydrophobic interactions, as previously reported^34^ (PDB 1LM8). Due to lower resolution in this region, the atomic details for ELOB are not fully visible; however, the overall fitting of secondary structure elements provided reliable information for the conformation of ELOB and its interaction with ELOC (Extended Data Fig. 4i). Notably, the C-terminal tail of ELOB is poorly defined in our structure. Superposition shows that the ELOB tail in the HIF-1α–pVHL–ELOC–ELOB structure clashes with ELOA in our structure (Extended Data Figs. 1g and 6c). In the HIF-1α–pVHL–ELOC–ELOB structure, the tail of ELOB folds back onto pVHL and is stabilized by this interaction. The clash between ELOA and the superposed ELOB excluded this stabilization, which could explain the less-defined ELOB tail in our structure.

Since Elongin can form a five-subunit complex with ubiquitin ligases CUL5–RBX2, we also modeled the five-subunit Elongin in complex with Pol II (Extended Data Fig. 1i). To this end, we superposed our structure and structures of Vif–CBFβ–ELOB–ELOC–CUL5 (PDB 4N9F)^32^ and the CUL5–RBX2 (PDB 6V9I)^31^ complexes (Extended Data Fig. 1h). No obvious additional contacts between CUL5–RBX2 and Pol II were observed in this model. Thus, although our structural results provide a basis to study how Elongin is involved in ubiquitylation, they do not offer immediate new insights into this aspect of Elongin function.

### Conserved Pol II–Elongin interaction

**Fig. 4:**
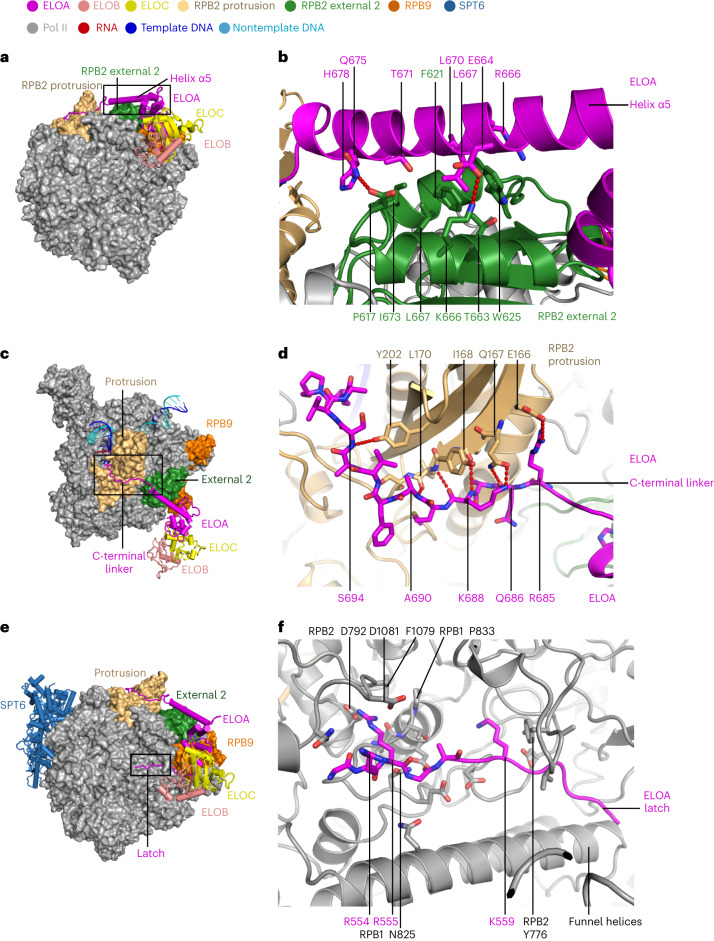
Pol II–Elongin interaction interfaces. **a**,**b**, Overview (**a**) and close-up view (**b**) of the ELOA–Pol II external 2 interface in structure 2. The region boxed in the overview is zoomed in and shown in **b**. Residues involved in the hydrophobic interactions are shown as sticks, including ELOA helix α5 residues R666, L667 and L670 and Pol II external 2 residues P617, F621, W625, T663, L667 and I673. **c**,**d**, Overview (**c**) and close-up view (**d**) of the ELOA–Pol II protrusion interface in structure 2. Hydrogen bonds are indicated by red dotted lines. Residues forming hydrogen bonds between ELOA and Pol II protrusion are shown as sticks. Hydrogen bond pairs, including the main chain of ELOA R685, Q686, K688, A690 and S694 and the side chain or main chain of RPB2 E166, Q167, I168, L170 and Y202, are shown. The coloring scheme is indicated by the colored dots. **e**,**f**, Overview (**e**) and close-up view (**f**) of the ELOA–Pol II interface near bridge helix and funnel helices in structure 1. In panel **f**, ELOB and ELOC are omitted for clarity. Residues at the interaction interface are shown as sticks. ELOA R555 inserts into a hydrophobic pocket formed by RPB1 P833, RPB2 F1079 and H793 and is in close proximity to acidic RPB2 residues D792 and D1081. ELOA K559 stacks onto RPB2 Y766.

Elongin binds to Pol II via its subunit ELOA, which forms three distinct interfaces with Pol II (Figs. 2a–c and 4a–f). ELOA binds the Pol II RPB2 domains external 2 and protrusion, and approaches the RPB9 C-terminal domain. ELOA binds to RPB2 external 2 via its helix α5 (residues 660–684), and to the protrusion via a C-terminal linker (residues 685–698) at the edge of the Elongin A superfamily domain, forming two interfaces with a total buried surface area of ~1,500 Å^2^ based on PISA estimation^33^ (Fig. 2a–c and Extended Data Fig. 6a). ELOA helix α5 interacts with the Pol II external 2 domain mainly via hydrophobic interactions. ELOA residues R666, L667 and L670 interact with a hydrophobic patch on external 2 formed by residues P617, F621, W625, T663, L667 and I673 (Fig. 4a,b).

The ELOA C-terminal linker interacts with the Pol II protrusion mainly via hydrogen bonds between main chain residues of ELOA (residues R685, Q686, K688, A690 and S694) and side chains or main chain atoms of RPB2 (residues E166, Q167, I168, L170 and Y202) (Fig. 4c,d). Additionally, the ELOA superfamily homology domain distantly contacts the RPB9 C-terminal domain. The ELOA residues on the RPB2–ELOA interface are highly conserved from human to worm, whereas the residues on the RPB9–ELOA interface are mostly variable (Extended Data Fig. 6a).

### An Elongin ‘latch’ approaching the Pol II active center

ELOA uses its latch element (residues 553–564, at the N-terminal end of the BC box) to contact the RPB1 bridge helix and funnel helices and form a third interface with Pol II (Figs. 2b,c and 4e,f and Extended Data Fig. 4l). The ELOA latch forms a wedge between the end of the bridge helix and the funnel helices and widens the groove between them. It contacts Pol II via hydrophobic and hydrophilic interactions, mainly contributed by ELOA residues R555 and K559 and surrounding RPB1 and RPB2 residues (Fig. 4e,f). The C-terminal half of the linker further contacts the funnel helices. Importantly, the binding sites of R555 and K559 locate around the base of the bridge helix, which is a key element for the regulation of Pol II activity^35^.

### Elongin elements required for elongation stimulation

**Fig. 5:**
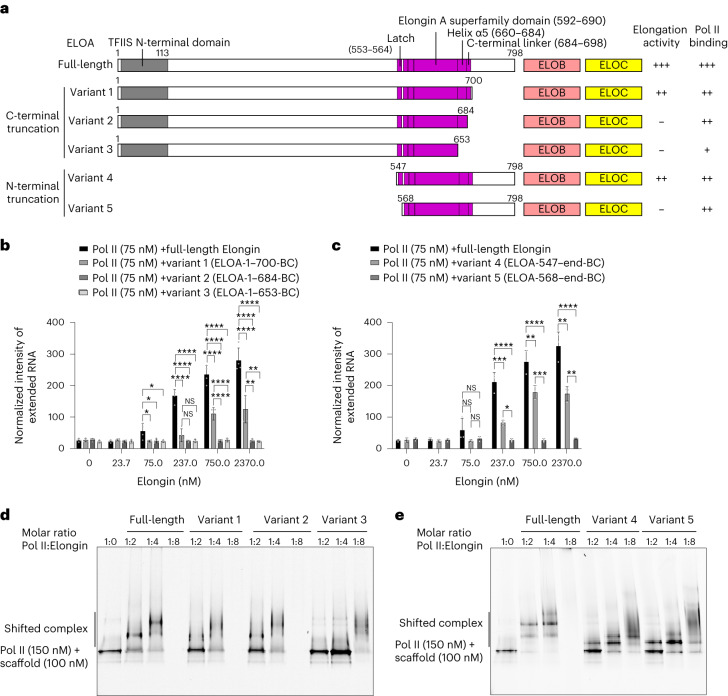
Pol II-binding elements of ELOA are required for the elongation stimulation activity of Elongin. **a**, Scheme of the Elongin variants containing ELOA truncated at the C or N terminus and full-length ELOB–ELOC. The elongation activity and Pol II-binding activity of each complex are summarized. A negative sign (−) indicates loss of activity. A positive sign (+) indicates the presence of activity, and the number of + signs indicates the strength of the activity. **b**,**c**, Quantification of the elongation-stimulatory activity of Elongin variants containing ELOA truncated at the C terminus (variants 1–3, **b**) or N terminus (variants 4 and 5, **c**). RNA extension assays were performed in the presence of increasing amounts of full-length Elongin or Elongin variants 1–3 (**b**, representative gels in Extended Data Fig. 7a) or Elongin variants 4 and 5 (**c**, Extended Data Fig. 7b). The extended RNA products are quantified as described in Methods. Bar diagram and error bars indicate the means and standard deviations of the integrated intensity of the extended RNA products from four (*n* = 4, **b**) and three (*n* = 3, **c**) independent experiments. The amount of extended RNA products in the presence of Elongin variants 1–3 is compared to that of full-length Elongin and variant 1. The amount of extended RNA products in the presence of Elongin variants 4 and 5 is compared to that of full-length Elongin and variant 4. The statistical significance *P* value was calculated with one-way analysis of variance (ANOVA) and indicated as pairs. NS (not significant), *, **, *** and **** indicate *P* > 0.05, *P* ≤ 0.05, *P* ≤ 0.01, *P* ≤ 0.001 and *P* ≤ 0.0001, respectively. The individual data points are shown as gray dots. (The exact *P* values and the unprocessed gel images are shown in the source data for this figure.) **d**,**e**, Electrophoretic mobility shift assays showing the Pol II-binding activity of Elongin variants with ELOA truncated at the C (**d**) or N terminus (**e**). Complex formation is indicated by upshifting of the elongation complex on Native PAGE. Panels **d** and **e** show representative gels of the three replicates of each panel. (Unprocessed gel images are shown in the source data for this figure.) Source data

**Extended Data Fig. 7:**
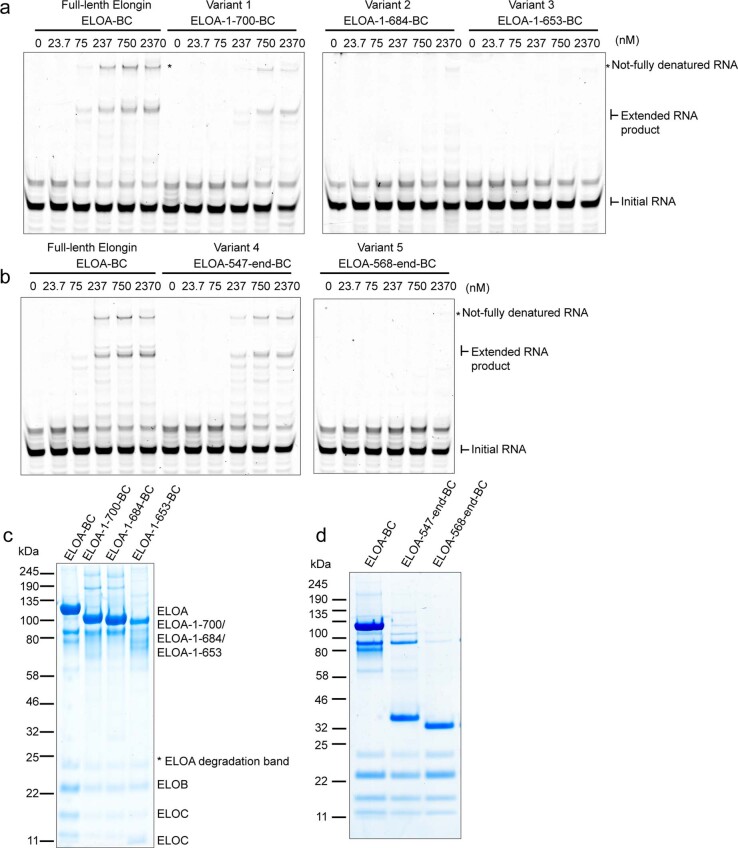
RNA extension assays with full-length Elongin and variants. **a**. Representative gels of RNA extension assays performed with full-length Elongin and variants containing ELOA N-terminal truncations (Variants 1-3, titration experiments). Increasing amount of full-length Elongin, ELOA-1-700-ELOBC (variant 1), ELOA-1-684-ELOBC (variant 2) or ELOA-1-653-ELOBC (variant 3) were incubated with pre-assembled Pol II elongation complex before starting transcription. The extended RNA products after 1 min incubation were analyzed on RNA gel. Quantification of the extended RNA products is shown in Fig. 5b. The unprocessed gel images are reported in Source Data Fig. 5. **b**. Representative gels of RNA extension assays performed with full-length Elongin and variants containing ELOA C-terminal truncations (variants 4-5, titration experiments). The experiments were performed as described in panel a. Quantification of the extended RNA products are shown in Fig. 5c. The unprocessed gel images are reported in Source Data Fig. 5. **c, d**. Analysis of Elongin and variants with SDS-PAGE. See unprocessed gels in Source Data Extended Data Fig. 7. The Elongin in use was from two purification batches. Each variant in use was from one batch of purification. The proteins were loaded side by side into the two SDS-PAGEs as representation of the protein quality. Source data

To determine which regions of Elongin are required for the elongation-stimulatory activity, we used our structure to design Elongin variant complexes (variants 1–5) lacking potential functional elements (Fig. 5a–c and Extended Data Fig. 7a–d). We first tested Elongin variants containing ELOA truncated from the C terminus (variants 1–3). Variant 1 contains the ELOA C-terminal linker and efficiently stimulated transcription elongation despite a slightly lower activity compared to the full-length Elongin. In contrast, the variants lacking the ELOA C-terminal linker (residues 685–700) that interacts with the Pol II protrusion (variant 2), or the C-terminal linker and helix α5 (variant 3), were unable to stimulate Pol II transcription (Fig. 5b and Extended Data Fig. 7a,c). This indicates that the ELOA C-terminal linker is required for the elongation stimulation activity of Elongin.

Next, we tested Elongin variants containing ELOA truncated from the N terminus (variants 4 and 5). Variant 5 lacks the ELOA latch (residues 547–567) and failed to stimulate transcription elongation, whereas the latch-containing variant 4 efficiently stimulated transcription elongation, although with lower activity relative to the full-length Elongin (Fig. 5c and Extended Data Fig. 7b,d). These data indicate that the ELOA latch and C-terminal linker are both required for the elongation-stimulatory activity of Elongin. Consistently, rat ELOA residues 521–680 (corresponding to human ELOA residues 548–707) were previously shown to be minimally required for elongation activity^26^. This indicates that the requirement of the ELOA latch and C-terminal linker in elongation stimulation is conserved between human and rat, and very likely also among other species, due to the high sequence conservation (Extended Data Fig. 6a).

To investigate whether the loss of activity of the Elongin variants was due to a loss of binding to Pol II, we performed electrophoretic mobility shift assays with Pol II elongation complex and Elongin variants (Fig. 5d,e). The results showed that all Elongin variants retained Pol II binding (Fig. 5d,e), despite their different abilities in elongation stimulation. Thus, the loss of elongation activity was not due to a loss of Pol II binding, but due to loss of changes in Pol II structure or mobility upon binding of Elongin variants.

### Allosteric stimulation of Pol II activity by Elongin

**Fig. 6:**
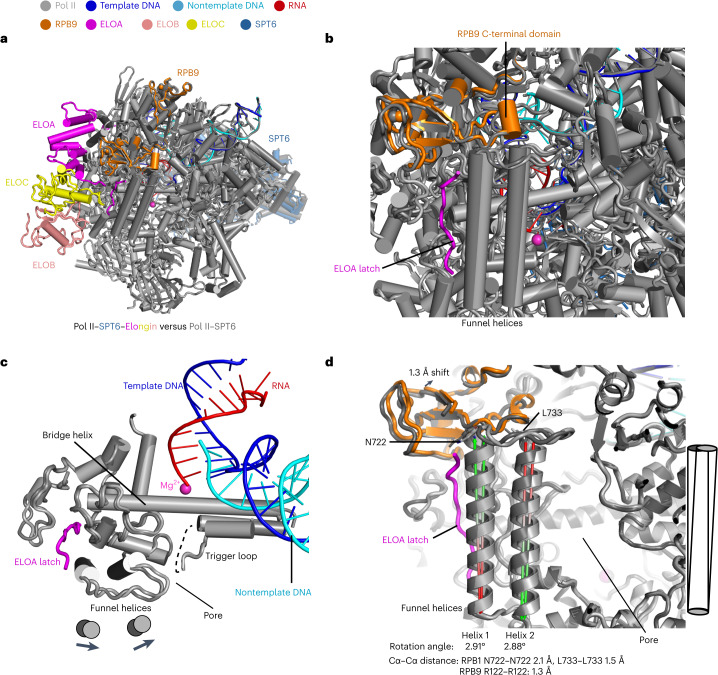
Elongin binding induces conformational changes in Pol II. **a**, Overview of the superposition between Pol II–SPT6–Elongin complex (structure 1) and Pol II–SPT6 complex (structure 4). Pol II is shown as cartoon presentation with cylinder helices. The color of protein subunits is indicated on the figure. **b**, Close-up view of the conformational change at Pol II RPB9 C-terminal domain and funnel domain. **c**, Close-up view of the conformational change around the bridge helix. Funnel helices move towards the trigger loop. **d**, Measurement of rotation angles and shifting distances of the funnel helices and RPB9. The angles between the funnel helices of Pol II–SPT6–Elongin and Pol II–SPT6 models were calculated in PyMOL with script ‘angle_between_helices’ ‘method=0’. RPB1 residues 696–721 are used for angle calculation of helix 1; residues 734–759 are used for angle calculation of helix 2. The Cα–Cα distance of RPB1 residues N722–N722 and L733–L733 from the two models are 2.1 Å and 1.5 Å, respectively, indicating movement of the funnel helices. The Cα–Cα distance of RPB9 R122–R122 from the two models is 1.3 Å, indicating movement of the RPB9 C-terminal domain.

**Extended Data Fig. 8:**
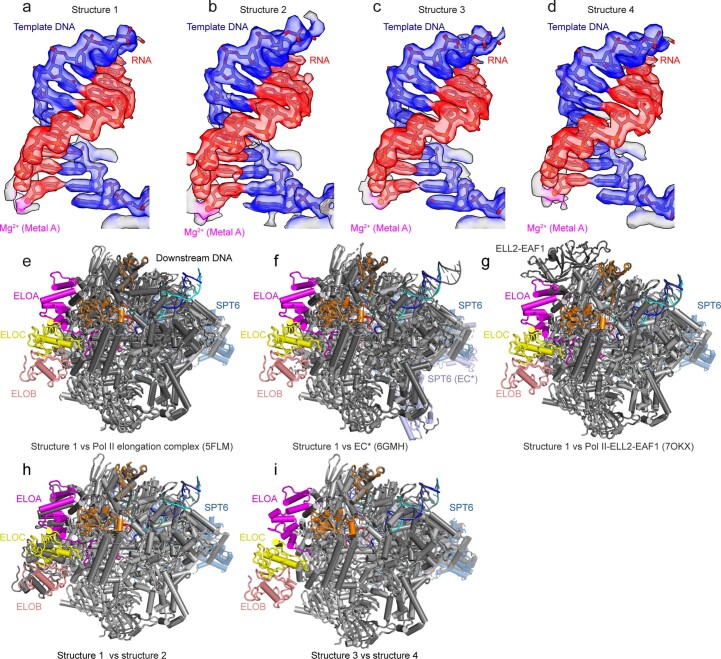
Elongin binding induces conformational changes in Pol II. **a-d**. Post-translocated states of structures 1-4. The DNA-RNA hybrid of each structure is shown as stick representation. The Mg^2+^ ion at the active site is shown as sphere representation. The cryo-EM map at this region is shown as surface representation. Template DNA, non-template DNA, RNA and Mg^2+^ are colored in blue, cyan, red and magenta, respectively. **e-g**. Superposition of the complete Pol II-SPT6-Elongin structure (structure 1) and Pol II elongation complexes by aligning the active site domain. **e**. structure 1 vs Pol II elongation complex (PDB: 5FLM)^28^. **f**. Structure 1 vs Pol II-SPT6-PAF complex (EC*, PDB: 6GMH^29^). **g**. Structure 1 vs Pol II-ELL2-EAF1(PDB: 7OKX)^36^. Pol II, ELOA, ELOB, ELOC and SPT6 in structure 1 are colored in light grey, magenta, salmon, yellow, and sky blue, respectively. Pol II elongation complex, EC*, Pol II-ELL2-EAF1 structures are colored in dark grey, except from SPT6 (in light blue) in EC*. In panel **f**, PAF subunits are omitted for clarity. **h**. Superposition of structure 1 and structure 2 (Pol II-Elongin lacking ELOA latch) by aligning the active site domain. The structures are shown as cartoon cylinder presentation. Color of structure 1 is as panel **e-g**. Pol II in structure 2 is in dark grey. **i**. Superposition of structure 3 (Pol II-SPT6-Elongin lacking ELOA latch) and structure 4 (Pol II-SPT6) by aligning the active site domain. In structure 3, Pol II, ELOA, ELOB, ELOC and SPT6 are colored in light grey, magenta, salmon, yellow and sky blue, respectively. Structure 4 is colored in dark grey.

To understand the molecular mechanism for the elongation stimulation activity of Elongin, we compared the conformation of Pol II with and without Elongin bound. In all our structures, Pol II elongation complexes are captured in an active, post-translocated state (Extended Data Fig. 8a–d). However, obvious conformational differences are observed in Pol II upon Elongin binding. Superposition of the complete Pol II–SPT6–Elongin structure (structure 1) and the Pol II–SPT6 structure (structure 4) showed that, in the presence of Elongin, the Pol II funnel helices shift towards the polymerase active center (Fig. 6a–d and Supplementary Video 1). The funnel helices rotate by ~3°, thereby narrowing the pore beneath the Pol II active site (Fig. 6c,d).

**Extended Data Fig. 9:**
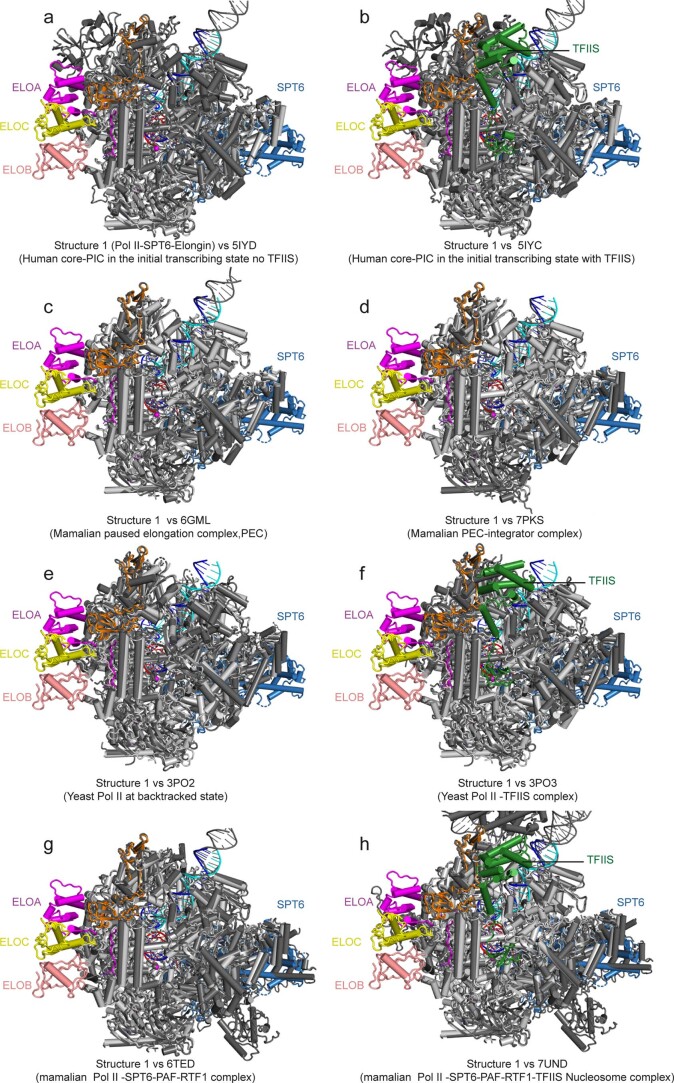
Elongin binding induces conformational changes in Pol II. **a**. Superposition of the complete Pol II-SPT6-Elongin structure (structure 1) and human core-PIC in the initial transcribing state without TFIIS present (PDB: 5IYD)^37^. **b**. Superposition of the complete Pol II-SPT6-Elongin structure (structure 1) and human core-PIC in the initial transcribing state TFIIS present (PDB: 5IYC)^37^. **c**. Superposition of the complete Pol II-SPT6-Elongin structure (structure 1) and the mammalian paused elongation complex (PEC, PDB: 6GML)^38^. **d**. Superposition of the complete Pol II-SPT6-Elongin structure (structure 1) and the mammalian PEC-integrator complex (PDB: 7PKS)^39^. **e**. Superposition of the complete Pol II-SPT6-Elongin structure (structure 1) and yeast Pol II at backtracked state(PDB: 3PO2)^41^. **f**. Superposition of the complete Pol II-SPT6-Elongin structure (structure 1) and yeast Pol II-TFIIS complex (PDB: 3PO3)^41^. **g**. Superposition of the complete Pol II-SPT6-Elongin structure (structure 1) and mammalian Pol II-SPT6-PAF-RTF1 complex (PDB: 6TED)^27^. **h**. Superposition of the complete Pol II-SPT6-Elongin structure (structure 1) and mammalian Pol II-SPT6-PAF-RTF1-TFIIS-nucleosome complex (PDB: 7UND)^48^. In all panels, ELOA, ELOC, ELOB, TFIIS and SPT6 are coloured in magenta, yellow, salmon, forest green and sky blue, respectively. Pol II in structure 1 is coloured in light grey and Pol II in other transcription complexes are coloured in darker grey.

Superpositions of structure 1 with the Pol II elongation complex (PDB 5FLM)^28^, the Pol II–PAF–SPT6 (EC*) complex^29^ or the Pol II–ELL2–EAF1 complex^36^ all show the same shift of the funnel helices in the presence of Elongin (Extended Data Fig. 8e–g). We also superposed structures 2 and 3, which contain Elongin including the ELOA C-terminal linker but lacking the latch, with the complete Pol II–SPT6–Elongin structure (structure 1) or the Pol II–SPT6 structure (structure 4). The superpositions show that Pol II in structures 2 and 3 adopts the same conformation as in structure 4 (Extended Data Fig. 8h,i). This indicates that the presence of the C-terminal linker in the absence of the ELOA latch does not induce a conformational change in Pol II. To further investigate the nature of the conformational change in Pol II, we superposed structure 1 with other transcription complexes, including the human core–PIC in the initially transcribing state without (PDB 5IYD) or with TFIIS (PDB 5IYC)^37^, the mammalian paused elongation complex (PDB 6GML)^27,38^, the active elongation complex Pol II–PAF1C–SPT6–RTF1 (PDB 6TED)^27^, the Pol II–DSIF–NELF–integrator complex (PDB 7PKS)^39^ and the Pol II–SPT6–PAF–TFIIS–nucleosome complex (PDB 7UND)^40^, as well as the yeast Pol II in an arrested, backtracked state (PDB 3PO2)^41^ and the reactivation intermediate (Pol II–TFIIS complex; PDB 3PO3)^41^ (Extended Data Fig. 9). These superpositions show that the conformational change in Pol II is only observed in the structure with Elongin containing the latch, confirming that the Elongin latch induces the conformational change.

As the ELOA latch is essential for Elongin activity, the conformational changes induced by latch binding may underlie Pol II stimulation by Elongin. Although the ELOA C-terminal linker is also required for the elongation stimulation activity of Elongin, its binding is insufficient to induce a conformational change in Pol II, but may stabilize the new Pol II conformation. Although it is unclear how the conformational change influences Pol II activity, it is possible that this occurs by restricting the mobility of the trigger loop. The trigger loop was shown to play a key role in the NTP addition cycle during transcription elongation^42^. Upon latch binding, the funnel helices move towards the trigger loop and could influence the dynamics of the trigger loop and thereby promote translocation. Elongin might stabilize Pol II in a post-translocated state and facilitate translocation. This is consistent with previous reports that Elongin stimulates transcription elongation by repressing transient pausing^2,6^.

### Incompatible binding of Elongin, PAF and SEC to Pol II

**Fig. 7:**
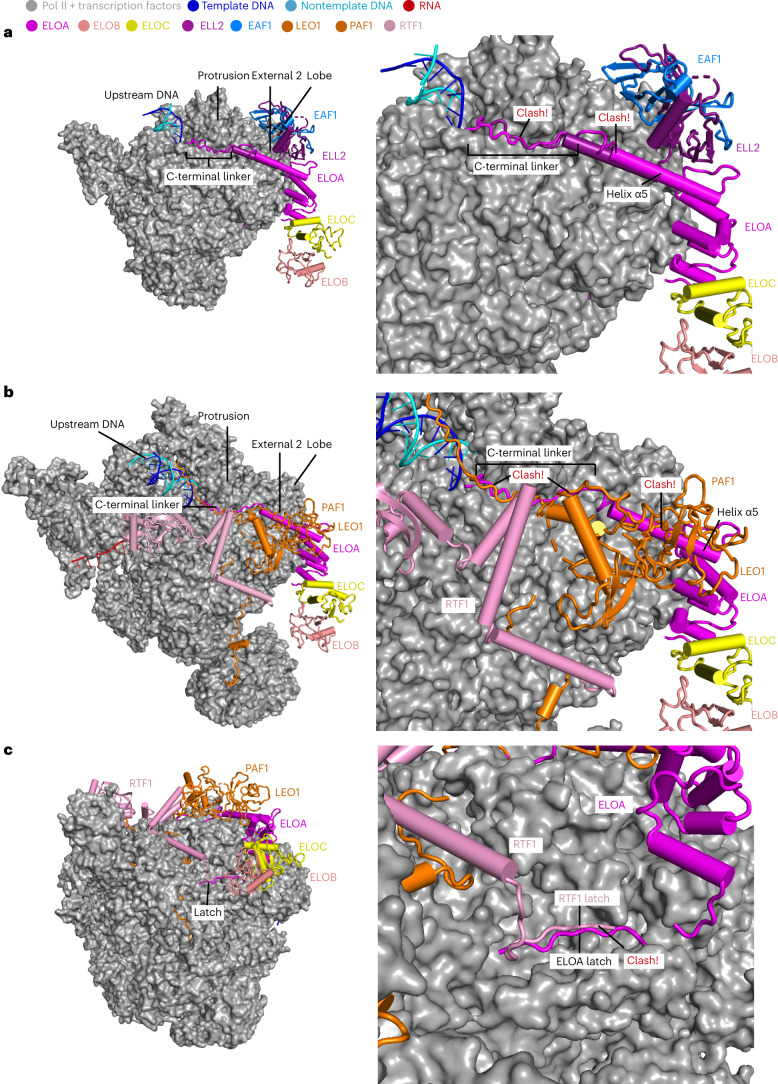
Elongin is inconsistent with binding of SEC and PAF complexes. **a**, Superposition of Pol II–ELL2–EAF1 structure (PDB 7OKX)^36^ onto Pol II–SPT6–Elongin structure (structure 1). Pol II is shown as surface representation in gray. ELL2–EAF1 and Elongin are shown as cartoon representation. The proteins are colored as indicated by the colored dots. **b**,**c**, Superposition of EC*–RTF1 (Pol II–DSIF–PAF–RTF1; PDB 6TED)^27^ structure onto Pol II–SPT6–Elongin structure (structure 1). PAF1–LEO1, RTF1 and Elongin are shown as cartoon representation. Other factors in EC* are shown as surface presentation. The color scheme is as in **a**.

As the ELL–EAF subcomplex of the super elongation complex (SEC) and the PAF (PAF1 complex)–RTF1 complex also stimulate transcription elongation, we compared the structures of the Pol II–SPT6–Elongin complex with the Pol II–DSIF–ELL2–EAF1 and the Pol II–PAF–RTF1–SPT6 (EC*) complexes^27,29,36^. Superposition of these complexes shows that ELOA, ELL2–EAF1 and PAF1–LEO1 bind to overlapping surfaces of Pol II (Fig. 7a,b). Both ELOA and ELL2–EAF1 bind to Pol II external 2 and protrusion domains; however, ELOA lacks the contact to the Pol II lobe domain, where ELL2–EAF1 dimerization domains bind (Fig. 7a). This difference could also explain why binding of Elongin did not induce conformational changes to the Pol II lobe, which was observed after binding of ELL2–EAF1 (Extended Data Fig. 8g)^36^. Similarly, ELOA clashes with the PAF1–LEO1 subcomplex of PAF on the Pol II surface at both protrusion and external 2 domains (Fig. 7b)^27^. The ELOA latch further clashes with the RTF1 latch near the funnel helices (Fig. 7c)^27^, suggesting that Elongin and RTF1 use similar mechanisms to stimulate Pol II elongation. In conclusion, these comparisons show that Elongin binding is structurally incompatible with binding of SEC, PAF or RTF1 to Pol II.

## Discussion

Here we prepared recombinant heterotrimeric human Elongin and determined three structures of the Pol II–SPT6–Elongin complex. Our work revealed the structure of trimeric Elongin, the interaction between Elongin and Pol II and conformational changes in Pol II that are induced by Elongin binding. A subsequent structure–function analysis further revealed that both the ELOA C-terminal linker and latch elements are required for the elongation-stimulatory activity of Elongin, and the latch is required for inducing conformational changes in Pol II. These results identified the elongation-stimulatory latch of Elongin as the key element for inducing a conformational change in Pol II that may be stabilized by the C-terminal linker.

**Extended Data Fig. 10:**
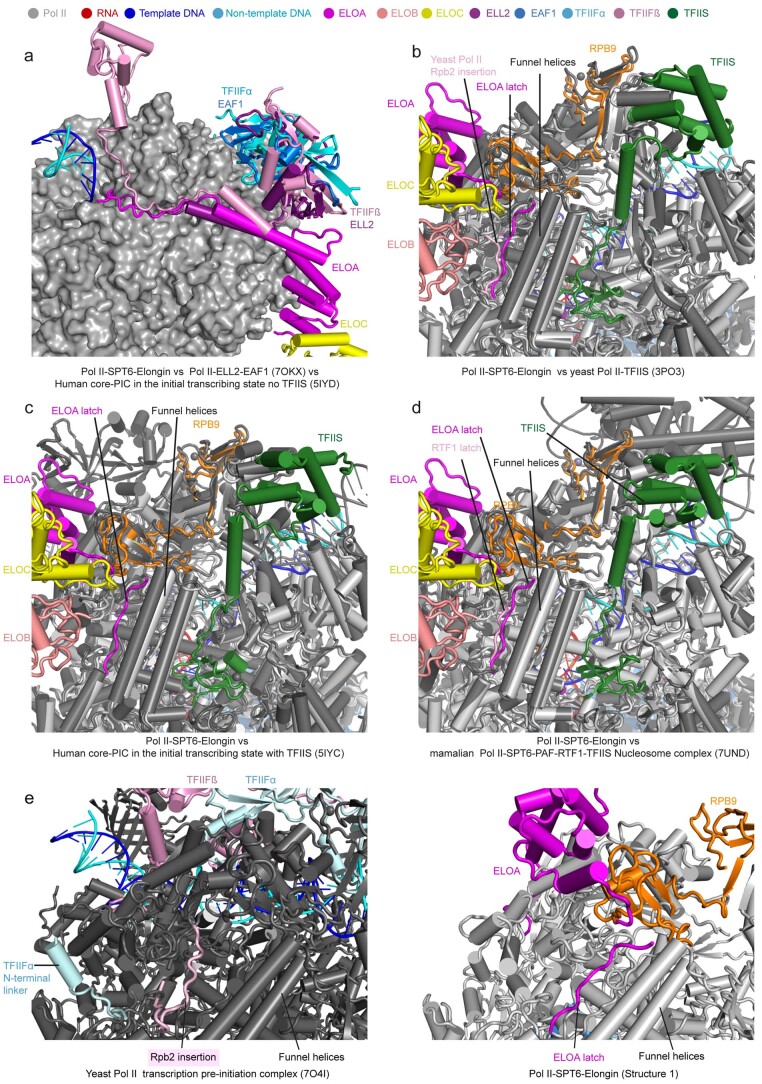
ELOA latch occupies similar Pol II region to the yeast Rpb2 insertion. **a**. Superposition of the structures of Pol II-SPT6-Elongin (structure 1), the Pol II-ELL2-EAF1 complex (PDB: 7OKX^36^) and the human core PIC in the initial transcribing state (PDB: 5IYD^37^). Proteins are coloured as indicated by coloured dots. **b**. Superposition of structures of Pol II-SPT6-Elongin (structure 1) and the yeast Pol II-TFIIS (PDB: 3PO3^41^) complexes. **c**. Superposition of the structures of Pol II-SPT6-Elongin (structure 1) and the human core PIC in the initial transcribing state with TFIIS complexes (PDB: 5IYC^37^). **d**. Superposition of the structures of Pol II-SPT6-Elongin (structure 1) and the mammalian Pol II-SPT6-PAF-RTF1-TFIIS nucleosome complex (PDB: 7UND^48^). **e**. Side-by-side comparison of the yeast Pol II - ‘Rpb2-insertion’ interaction and the Pol II-ELOA interaction (closeup view). Left panel: Pol II transcription pre-initiation complex with initial transcription bubble (PDB: 7O4I^43^); Right panel: the Pol II-SPT6-Elongin complex (structure 1). Yeast TFIIFα N-terminal linker extends towards the funnel helices, but does not reach the ELOA latch binding site, which is occupied by yeast Rpb2 insertion in the yeast pre-initiation complex.

Comparisons to previous structures suggest that three contact sites on Pol II are required for multisubunit elongation factors to stimulate Pol II. First, Elongin, TFIIF, the ELL–EAF subcomplex of SEC and the PAF1–LEO1 subcomplex of PAF all dock to the external 2 domain of the Pol II subunit RPB2 (refs. ^29,36,37^). Second, a linker winds around the Pol II protrusion and reaches the Pol II upstream cleft in all four complexes. Third, Elongin and RTF1, but not ELL–EAF and TFIIF, contain a latch element that binds near the end of the Pol II bridge helix. In contrast, ELL–EAF and TFIIF contact the Pol II lobe domain (Extended Data Fig. 10a). Although the resulting conformational changes in Pol II differ for Elongin and SEC, both factors likely influence the mobility of Pol II elements in the active center, and thereby stimulate its activity. Note that Pol II conformational changes were not captured upon PAF–RTF1 binding. This may have been due to the high flexibility of the RTF1 latch, and thus Elongin and RTF1 may use similar mechanisms. As was discussed for the RTF1 latch^27^, the ELOA latch also occupies the same region of Pol II as the yeast loop insertion at the external 1 domain of Rpb2 (residues 714–731; Extended Data Fig. 10b,e)^41,43^. Yeast TFIIF contains an N-terminal linker (residues 17–35) that extends to the direction of the Rpb2 insertion, but does not further extend to the funnel helices (Extended Data Fig. 10e)^43^. On the basis of these structural similarities and differences, we suggest that in terms of the mechanism for elongation stimulation, Elongin resembles RTF1 and TFIIF resembles ELL–EAF.

Our structural comparisons suggest that Elongin, SEC, PAF and TFIIF cannot bind simultaneously to the Pol II elongation complex. Consistent with this, published data suggest different functions for these factors in vivo. Elongin binds near promoter regions, pause sites of long genes and the termination site of short genes^44,45^, whereas SEC regulates pause release of heat shock response genes^46^ and PAF regulates Pol II processivity^47^. With respect to the function of these different elongation factors, it is important to recall that Elongin, ELL–EAF and TFIIF suppress TFIIS-stimulated cleavage of nonarrested Pol II transcripts^2^, possibly by controlling the orientation of the 3′ ends of the nascent transcripts or by blocking the binding of TFIIS to Pol II, as suggested by the Conaway laboratory^2^. Our superpositions of structures of the Pol II–Elongin–SPT6 with Pol II elongation complexes containing TFIIS reveal no clash between elongation factors and TFIIS^37,48^ (Extended Data Fig. 10b–d), suggesting that the elongation factors do not interfere with TFIIS binding, but rather may modulate the ability of TFIIS to reach or remodel the catalytic center of Pol II.

Finally, our data provide a basis for further analysis of the role of Elongin in Pol II degradation. The trimeric Elongin complex interacts with CUL5–RBX2, forming a five-subunit ubiquitin ligase that targets RPB1 in elongation-stalled Pol II^22,23^. Although our modeling did not suggest a direct contact between CUL5–RBX2 and Pol II, alternative conformations may be induced in vivo in the presence of post-translational modifications or binding partners. Future directions therefore include further analysis of how Elongin switches from an elongation factor to a ubiquitin ligase^49,50^ and how Elongin regulates transcription elongation in vivo^44,45^.

## Methods

### Cloning and protein expression

To express the heterotrimeric Elongin complex, Elongin A (ELOA, UniProt AC: Q14241) Elongin B (ELOB, UniProt AC: Q15370) and Elongin C (ELOC, UniProt AC: Q15369) DNA was amplified and cloned into 438B vectors, respectively, resulting in an N-terminal His-tagged ELOA with a TEV protease cleavage site between His-tag and ELOA and nontagged ELOB and ELOC. ELOA, ELOB and ELOC were subcloned into one plasmid using LIC subcloning strategy (MacroLab)^51^ for coexpression in insect cells. Elongin variants 1 (ELOA-1–700-ELOB-ELOC), variant 2 (ELOA-1–684-ELOB-ELOC), variant 3 (ELOA-1–653-ELOB-ELOC) and variant 5 (ELOA-568–end-ELOB-ELOC) with ELOA truncations were cloned in the same way. The ELOA in variant 4 (ELOA-547–end-ELOB-ELOC) was cloned into the 438C vector, which provides an N-terminal His-MBP-TEV tag before ELOA, and subcloned together with full-length ELOB and ELOC as described for the full-length Elongin. Primers and templates used in cloning Elongin are listed in Supplementary Data 2.

To express proteins in insect cells, bacmids were generated using DH10αEMBacY cells and transfected into Sf9 cells (Thermo Fisher Scientific, catalog no. 11496015) for V_0_ virus production, which were used to infect Sf21 cells (Expression Systems, catalog no. 94-003F) to produce V_1_ virus. Large-scale expression was performed in Hi5 cells (Expression Systems, catalog no. 94-002F). Hi5 cells were collected by centrifugation and resuspended in lysis buffer (50 mM HEPES pH 7.5, 400 mM NaCl, 30 mM imidazole, 10% glycerol and 1 mM DTT) supplemented with 1× protease inhibitor (1 mM PMSF, 2 mM benzamidine, 0.6 μM leupeptin and 2 μM pepstatin) before being flash frozen and stored at −80 °C.

### Protein purification

The full-length Elongin and variants 1–3 were purified from 2–4 l of Hi5 cells. Cells were lysed by sonication and cleared by centrifugation at 25,000 r.p.m. (23,059*g* at *r*_av_) for 30 mins with an A27 rotor (Thermo Scientific) and at 45,000 r.p.m. (158,000*g* at *r*_av_) with a Type 45 Ti rotor (Beckman) at 4 °C. Clear lysate was filtered through a 0.8 µm filter and loaded onto a 5 ml HisTrap HP column (Cytiva, nickel column), which was pre-equilibrated with lysis buffer. The nickel column was sequentially washed with lysis buffer, high salt buffer (50 mM HEPES pH 7.5, 800 mM NaCl, 30 mM imidazole, 10% glycerol and 1 mM DTT) and lysis buffer. The complex was eluted from the nickel column with a linear imidazole gradient from 30 mM to 500 mM. The eluate of the nickel column was cleaved with TEV protease to remove the N-terminal His-tag of ELOA, while dialyzing to the dialysis buffer (25 mM HEPES pH 7.4, 250 mM NaCl, 10% glycerol and 1 mM DTT). The cleaved complex was loaded onto tandem 5 ml HisTrap HP–HiTrap SP HP columns (Cytiva), which were pre-equilibrated with dialysis buffer (25 mM HEPES pH 7.4, 250 mM NaCl, 10% glycerol and 1 mM DTT). After washing with dialysis buffer, the protein was eluted with a linear salt gradient (250 mM–1 M NaCl) and further polished by gel filtration using Superdex 200 10/300 increase column (GE Healthcare). The complex was concentrated with a 10 or 30 kDa cut-off concentrator (Amicon), in buffer containing 20 mM HEPES pH 7.4, 400 mM NaCl, 10% glycerol and 1 mM DTT and stored at −80 °C.

Elongin variant 4 was in-batch purified using a gravity flow column (BioRad) containing 10 ml amylose resin (Biolabs), followed by cleavage of the His-MBP tag by TEV protease, tandem 5 ml HisTrap HP nickel–HiTrap HP heparin columns and gel filtration using a Superdex 75 10/300 increase column (GE Healthcare). Elongin variant 5 was purified in the same way as variant 4, except for substituting the MBP batch affinity step by affinity purification with a 5 ml HisTrap HP nickel column (GE Healthcare).

Pol II was purified from pig thymus^52^ and human SPT6 was expressed and purified from insect cells, respectively, as previously described^29^.

### Cryo-EM sample preparation

The Pol II–SPT6–Elongin complex was assembled onto a TAR RNA–DNA scaffold (Extended Data Fig. 2a) and incubated with SPT6 and trimeric Elongin in the presence of protein kinase complex P-TEFb-5aa-TAT and ATP to phosphorylate Pol II and elongation factors. The phosphorylation step was introduced to potentially enhance the Pol II–Elongin interaction, as it was shown that ELOA colocalizes with the hyperphosphorylated form of Pol II in vivo^44,53^. P-TEFb was fused with TAT to increase its stability^54,55^. The assembled complex was purified and cross-linked with glutaraldehyde by GraFix^56^. The complex was deposited onto cryo-grids and used for screening and data collection. Details are described below.

To assemble the Pol II–SPT6–Elongin complex, Elongin and SPT6 were mixed with pre-assembled Pol II–scaffold elongation complex, phosphorylated with P-TEFb and further purified by GraFix^56,57^ in a glycerol gradient (light solution: 20 mM HEPES pH 7.5, 150 mM NaCl, 3 mM MgCl_2_, 1 mM DTT and 10% glycerol; heavy solution: 20 mM HEPES pH 7.5, 150 mM NaCl, 3 mM MgCl_2_, 1 mM DTT and 30% glycerol, 0.1% glutaraldehyde) by centrifugation at 33,000 r.p.m. (111,845*g* at *r*_av_) for 18 h at 4 °C (Extended Data Fig. 2a–c). The gradient was aliquoted to 200 µl fractions and quenched by adding Asp (final concentration 8 mM) and Lys (final concentration 10 mM). The fractions were then analyzed with Native PAGE (Invitrogen).

Peak fractions were deposited onto continuous carbon (∼2.1 nm in thickness) and transferred onto Quantifoil R3.5/1 200 Cu mesh cryo-grids^56^. The grids were blotted double-sided, with a blot force of 5 for 1 s and flash frozen in liquid ethane with Vitrobot Mark IV at 4 °C and humidity of 100% (Thermo Fisher). The cryo-grids were screened on a 200 kV Glacios microscope. The best sample was saved for high-resolution data collection on a Titan Krios. Detailed complex assembly steps are described below.

The scaffold in use is a HIV-TAR RNA scaffold, consisting of a 5′ biotin-labeled nontemplate JH_Fix2 DNA (NT-DNA) 5′-/biotin/-CCATTGAGAGCGGCCCTTGTGTTCAGGAGCCAGCAGGGAGCTGGGAGC, a template JH_Fix2 DNA (GCTCCCAGCTCCCTGCTGGCTCCGAGTGGGTTCTGCCGCTCTCAATGG) and a 5′-FAM-labeled TAR RNA 5′-6-FAM- UUAAGGAAUUAAGUCGUGCGUCUAAUAACCGGAGAGGGAACCCACU (Extended Data Fig. 2a)^29^. The TAR RNA was refolded by sequential incubation at 95 °C for 3 min and on ice for 10 min in RNA folding buffer (20 mM HEPES pH 7.5, 100 mM NaCl, 3 mM MgCl_2_ and 10% glycerol). Template DNA and refolded RNA were annealed by incubating at 45 °C for 5 min and gradually decreasing the temperature from 45 °C to 25 °C (1 °C min^−1^) in RNA folding buffer. Pol II–scaffold complex was assembled by sequentially incubating 88 picomoles of Pol II with 146 picomoles DNA–RNA hybrid and 146 picomoles NT-DNA at 30 °C for 10 mins. Pol II–scaffold, SPT6 (360 pmoles) and Elongin (440 pmoles) were mixed and phosphorylated by incubating with 1 µM P-TEFb-5aa-TAT and 1 mM ATP at 30 °C for 30 min in buffer containing 20 mM HEPES pH 7.5, 175 mM NaCl, 4% glycerol, 3 mM MgCl_2_ and 1 mM DTT (final volume 70 µl), followed by additional 30 mins incubation on ice.

### Cryo-EM data collection and processing

Cryo-EM data for the Pol II–SPT6–Elongin complex was collected on a Titan Krios transmission electron microscope (TEM) (Thermo Fischer Scientific) operated at 300 keV, equipped with a K3 summit direct detector (Gatan) mounted behind a GIF Quantum LS energy filter (Gatan). The data collection was performed in EFTEM mode with a slit of 20 eV. In total, 35,579 micrographs were recorded with a pixel size of 1.05 Å/px in nonsuper-resolution counting mode using SerialEM^58^. A total exposure of 40.09 e^−^/A^2^ was accumulated over 2.9 s and fractionated into 40 movie frames.

Motion correction and contrast transfer function (CTF) estimation and particle picking were carried out with Warp^59^. Particles were subjected to two-dimensional (2D) classification, ab initio reconstruction and heterogeneous refinement in CryoSPARC^60^. The heterogeneous refinement class containing extra density for Elongin was refined and used as input reference for data processing in Relion^61^.

About 7.3 million Warp-picked particles were re-extracted in Relion with 4× binning and a pixel size of 4.2 Å. Then, 2D and 3D classifications were performed to remove damaged particles and contaminants. The remaining 4,110,669 high-quality particles were re-extracted with the original pixel size of 1.05 Å and subjected to a global 3D refinement using the cryoSPARC map as reference model. To sort out particles that contain the Elongin complex, focused 3D classification without image alignment (four classes, *T* = 200) was performed with a local mask enclosing the Elongin region, on the basis of the global 3D refinement. About 18% of the high-quality particles contained the Pol II–SPT6–Elongin complex, while ~68% of that contained only the Pol II–SPT6 complex.

Particles containing Elongin were subjected to global 3D refinement, Bayesian polishing and CTF refinement, yielding a consensus map of the Pol II–SPT6–Elongin complex at 2.64 Å (Fourier shell correlation (FSC) = 0.143, map 1). However, due to occupancy and flexibility issues, the resolution of Elongin and SPT6 and upstream DNA was much lower (Extended Data Fig. 4a). The particles were further classified with an Elongin local mask to increase Elongin occupancy. Two classes (classes 3 and 4, maps 2 and 3) showed density for the full Elongin complex, which showed different orientation relative to Pol II. The class with higher local resolution for Elongin (class 3, map 2) was further classified to obtain higher resolution maps for the flexible regions with local masks around Elongin, upstream DNA and SPT6 (Extended Data Fig. 3c).

Focused 3D classification with the Elongin local mask yielded four classes. Classes 1, 3 and 4 contained density for Elongin, while class 2 only showed density for ELOA. The particles in classes 1, 3 and 4 were combined and refined globally (map 4, 2.81 Å) and with local mask around Elongin, Pol II core–ELOA and Pol II stalk, resulting in focused maps for these regions at 3.64 Å (map 5), 2.69 Å (map 6) and 3.67 Å (map 7), respectively (Extended Data Fig. 3c). Similarly, the focused map for upstream DNA (map 9, 5.6 Å) was obtained by focused classification using a local mask at the upstream DNA region, followed by global and focused refinement of the class with highest upstream DNA occupancy (class 2). A composite map (composite map 1), which combined focused maps for Elongin (map 5), Pol II core (map 6), stalk (map 7) using map 4 as the consensus map, was used to build the high-resolution Pol II–Elongin model (structure 2).

Focused classification with the SPT6 mask yielded four classes. Particles in class 1 have very low occupancy for SPT6, while classes 2–4 showed SPT6 at different orientations relative to Pol II. The particles in classes 2–4 were combined, refined globally and then locally with a SPT6-stalk mask, resulting in an overall map for Pol II–SPT6-Elongin at 2.86 Å (map 10) and a focused map for SPT6-stalk at 4.2 Å (map 11) **(**Extended Data Fig. 3c). Maps 10 and 11 were combined as composite map 2, which was used to build the Pol II–SPT6–Elongin model (structure 3).

Additionally, extra density for an ELOA linker at the N terminus of the BC box, here termed the ‘latch’, is visible in the unsharpened map 4 at *B* = 0. To improve the occupancy for the ELOA latch, particles belonging to map 3 and map 4 were combined and classified with a local mask around Pol II funnel helices. For this, 23% of the particles contained the extra density for ELOA latch (class 2), while 68% of the particles lacked the extra density (class 3). The particles in class 2 were refined globally, resulting in a global map of 3.05 Å (map 12). Map 12 was used for building the Pol II–SPT6–Elongin model including the ELOA latch (structure 1).

Particles that contained only the Pol II–SPT6 complex were sorted first by global 3D classification to remove bad particles and then focused classified with a local mask for SPT6. Particles in classes 2–4 were combined, refined globally and then focused classified, resulting in four classes that contained SPT6 in different orientation to Pol II. The class with highest SPT6 resolution was refined globally (map 14) and locally with a SPT6-stalk mask, resulting in a focused map for SPT6 at 3.64 Å (map 15).

To build the Pol II–SPT6 model (structure 4), the focused map for SPT6 (map 15) was combined with the global map 14 forming the composite map 3, which represents one of the many states of Pol II–SPT6 in the data.

### Model building and refinement

To build the high-resolution Pol II–Elongin model lacking the ELOA latch (structure 2), Pol II from the Pol II–ELL2–EAF1 model (PDB 7OKX)^36^ was placed into map 4 in Chimera^62^. The starting model of the Elongin complex was generated by AlphaFold2 (ref. ^63^) using the C-terminal region of ELOA (residues 531–798), ELOB and ELOC as input sequences. Pol II and Elongin models were fitted into the consensus map (map 4) in Chimera and manually adjusted in Coot^64^. The Elongin model was built into focused refinement map 5. Upstream DNA was placed into map 9, which was prefitted into map 4. The resulting model was refined against composite map 1 with real-space refinement in Phenix^65,66^. The DNA and RNA sequences were unambiguously assigned according to the high-resolution map at the active site and DNA–RNA hybrid (Extended Data Fig. 4g). A ten-base pair DNA–RNA hybrid was observed in the structure (post-translocated position, –1) (Extended Data Fig. 4g).

Manual rebuilding in Coot^64^ and real-space refinement in Phenix^66^ were performed iteratively to improve model geometry and model-to-map fitting, using the following refinement strategies: minimization global, local grid search and ADP refinement with secondary structure and Ramachandran restraints. This resulted in a high-resolution model consisting of Pol II and Elongin (Table 1).

To build the Pol II–SPT6–Elongin model lacking ELOA latch (structure 3), the Pol II–Elongin model (structure 2) and the SPT6 model from 7OOP (ref. ^67^) were fitted as a rigid body into the composite map 2, and refined by treating SPT6 and Pol II–Elongin as separate rigid bodies against composite map 2 in Phenix^66^.

To build the Pol II–SPT6–Elongin model with ELOA latch (structure 1), Pol II from structure 2 was adjusted against sharpened map 12 (*B* = −49). The ELOA latch was built into map 12 at lower *B* factor (*B* = −20). We tested different sequence register possibilities for the ELOA latch (residues 553–564), the current model fits best into the cryo-EM map. The bulky residues (R555 and K559) with defined density were used as register markers. Pol II, SPT6 and global domains of Elongin were finally fitted into local resolution filtered map 12 (Extended Data Fig. 4m) and refined as rigid bodies in Phenix.

In addition to the Pol II–SPT6–Elongin structures, we built and refined a Pol II–SPT6 model (structure 4) from a reconstruction that lacked Elongin (Table 1). To this end, we removed Elongin from our refined structure and fitted the resulting model into map 14 and manually adjusted it in Coot^64^. The flexible Pol II stalk–SPT6 region was built into a stalk-SPT6 focused map (map 15). The two models were fitted into composite map 3 and refined with real-space refinement in Phenix^66^. For all structures, model-to-map fit FSC curves and local resolution estimates are shown in Extended Data Fig. 4a–d. Final statistics for the structures are provided in Table 1. Structural figures and movies were generated in Chimera^62^, Chimera X^68^ and PyMOL (The PyMOL Molecular Graphics System, v.2.4.1, Schrödinger, LLC).

### Cross-linking mass spectrometry

A 90 pmol portion of Pol II–SPT6–Elongin complex was assembled as described for the cryo-EM sample. The assembled complex was cross-linked in-batch by incubating with 3 mM bis(sulfosuccinimidyl)suberate (BS3; Thermo Scientific) at 5 °C for 1 h and quenched with 100 mM Tris pH 7.6. The cross-linked sample was purified by 4 ml 10–30% glycerol density gradient ultracentrifugation, at 36,000 r.p.m. (133,104*g* at *r*_av_) for 18 h at 4 °C using an SW Ti60 rotor (Beckmann). The gradient was fractionated to 200 µl fractions and checked on Native PAGE (Invitrogen). Fractions 14–19 contained the target complex and were pelleted using an S150AT rotor (Thermo Fisher Scientific).

Cross-linked complexes were solubilized with 4 M urea in 50 mM ammonium bicarbonate (pH 8.0), reduced with DTT and alkylated with iodoacetamide. After dilution to 1 M urea with 50 mM ammonium bicarbonate, cross-linked complexes were digested with trypsin (Promega) in a 1:20 enzyme-to-protein ratio (w/w) at 37 °C overnight. Peptides were reverse-phase extracted using SepPak Vac tC18 1cc/50 mg (Waters) and eluted with 50% acetonitrile (ACN)/0.1% TFA. The eluate was lyophilized. Lyophilized peptides were dissolved in 40 µl 2% ACN/20 mM ammonium hydroxide and separated on basic pH reverse phase (bRP) using an xBridge C18 3.5 µm 1 mm × 150 mm column (Waters) with a 4–36% ACN gradient over 45 min at a flow rate of 60 µl min^−1^. One-minute fractions of 60 µl were collected, pooled in a step of 12 min (resulting in 12 pooled fractions total), vacuum dried and dissolved in 5% ACN/0.1% TFA for subsequent uHPLC–ESI–MS/MS analysis.

bRP fractionated peptides were measured in triplicate on an Orbitrap Exploris 480 (Thermo Fisher Scientific). The mass spectrometer was coupled to a Dionex UltiMate 3000 uHPLC system (Thermo Scientific) with a custom 35 cm C18 column (75 µm inner diameter packed with ReproSil-Pur 120 C18-AQ beads, 3 µm pore size, Dr. Maisch). MS1 and MS2 resolutions were set to 120,000 and 30,000, respectively. Only precursors with a charge state of 3–8 were selected for MS2. Protein–protein cross-links were identified by pLink2.3.11 search engine (pfind.org/software/pLink) according to the recommendations of the developer^69,70^.

The cross-links were mapped onto the Pol II–SPT6–Elongin model with Chimera^62^ and XlinkAnalyzer^71^. The peptides with count of spectrums (CSMs) larger than 4 and with FDR 0.01 were mapped to the model (Extended Data Fig. 5b–d). Among the 172 unique cross-links mapped, 91.9% satisfied the Cα–Cα distance <30 Å criterion, which is bound by the maximum length of the BS3 cross-linker (Extended Data Fig. 5a). This indicates a good agreement of the cross-linking mass spectrometry data and cryo-EM modeling.

### RNA extension assays

RNA extension assays were performed to test the activity of Elongin and its variants on Pol II transcription elongation with a set of A-less JUNB scaffold containing template DNA (JUNB-A-less-T-DNA) GAAACCCCCAGCACCCAGCACCCAGCAGGCACCGAGGCTGGCCTGGCCGCTCTCAAGGTCCCA, 5′-biotin-labeled nontemplate DNA (JUNB-A-less-NT-DNA) 5′-/biotin/-TTTTTTGGGACCTTGAGAGCGGCCAGGCCAGCCTCGGTGCCTGCTGGGTGCTGGGTGCTGGGGGTTTC and a short 5′-FAM-labeled RNA primer (JUNB U-RNA) 5′-6-FAM-UUUUUUUCAGGCCAGCC, as previously described (Extended Data Fig. 1a)^27^. Oligonucleotides were purchased from Integrated DNA Technologies. Template DNA and RNA were annealed by first incubating at 95 °C for 5 min and decreasing the temperature from 95 °C to 30 °C in 1 °C min^−1^ steps in RNA folding buffer (20 mM HEPES pH 7.5, 100 mM NaCl, 10% glycerol and 3 mM MgCl_2_). Briefly, the transcription reactions contained 75 nM Pol II, 50 nM RNA–DNA hybrid, 50 nM nontemplate DNA, 100 mM NaCl, 20 mM Na-HEPES pH 7.5, 3 mM MgCl_2_, 1 mM DTT, 4% glycerol and 10 µM NTPs (CTP, GTP and UTP) and various concentrations of elongation factors. Pol II was first assembled with the template DNA–RNA hybrid, followed by the addition of the nontemplate DNA. The complex was incubated at 30 °C for 10 min, while shaking (300 r.p.m.). Then, 4× assay buffer and water were added to the Pol II–scaffold complex to obtain the final assay conditions. Pol II and elongation factors were pre-incubated at 30 °C for 15 min before starting RNA extension to allow complex formation.

Transcription assays were performed at 30 °C. For time-course experiments, samples were taken at time points of 0 s, 10 s, 30 s, 60 s, 120 s, 300 s and 600 s. For the titration experiments, Elongin and variants were added at a final concentration between 0 and 2,370 nM. Transcription was stopped after 1 min incubation with NTPs by adding 2× quenching buffer (1× TBE buffer, 20 mM EDTA pH 8.0 and 6.5 M urea) in 1:1 ratio. The RNA products were analyzed on 20% acrylamide–urea gels. An 8 µl sample was loaded to each lane. The gels were run at 300 V for ~90 min in 0.5× TBE buffer. RNA signal was detected by scanning the fluorescence of the 5′-FAM label on the RNA primer with Typhoon FLA9500 (GE Healthcare) using a 473 nm wavelength laser at photomultiplier tube (PMT) of 750. Example RNA gel images are shown after subtracting overall gel background and enhancing contrast level over the whole gel. Only a fraction of the initial RNAs were extended in our assays, possibly due to the inefficiency of functional Pol II–scaffold assembly.

To quantify the extended RNA products, the integrated intensity of the extended RNA band and a normalization RNA band, the 5th band counting from the bottom of the gel, which is part of the synthesized RNA, in each lane was measured using a box with dimensions 0.42 × 0.33 cm^2^ (84 × 66 pixels) and 0.42 × 0.25 cm^2^ (84 × 50 pixels), respectively, after subtraction of overall gel background in ImageJ (Fiji). The normalized intensity (Int norm) of extended RNA was calculated by dividing the integrated intensity of extended RNA (Int E) with the integrated intensity of the normalization RNA band (Int N) followed by multiplying by 100 in Excel (Microsoft) (Int norm = Int E/Int N × 100). For time-course experiments, the normalized intensity of extended RNA products at 0 s (*t* = 0) was further subtracted from all later time points (*x*) before plotting (Int norm _(*t*=*x*)_ = (Int E/Int N_(*t*=*x*)_ – Int E/Int N_(*t*=0)_) × 100).

For titration experiments, statistical significance *P* values of differences between the experimental groups and the chosen ‘control’ were calculated in GraphPad Prism v.9 using ordinary one-way ANOVA. NS, *, **, *** and **** indicate *P* > 0.05, *P* ≤ 0.05, *P* ≤ 0.01, *P* ≤ 0.001 and *P* ≤ 0.0001, respectively. The mean and standard deviation from at least three independent experiments were calculated and plotted against time (time course) or against the concentration of protein factors (titration) in GraphPad Prism v.9 (see the source data for Figs. 1 and 5 and Extended Data Fig. 1).

### Electrophoretic mobility shift assays

Electrophoretic mobility shift assays were performed by incubating pre-assembled Pol II–scaffold complex and Elongin variants on ice for 1 h, followed by analysis of the complexes with Native PAGE (Invitrogen). The scaffold contained the same template DNA and nontemplate DNA as that in the assembly scaffold (Extended Data Fig. 2a) used for cryo-EM study and a 5′-FAM-labeled 18-mer TAR RNA (5′-6-FAM-UAACUAGGGAACCCACU). This shorter 18-mer RNA was used to avoid unspecific interaction between protein factors and the exposed RNA outside of the Pol II RNA exiting channel. The final reaction contained 150 nM Pol II, 100 nM scaffold and 150 nM to 1.2 µM Elongin variants in 12 µl reaction and in buffer containing 20 mM Na-HEPES pH 7.5, 175 mM NaCl, 10% glycerol and 1 mM DTT. Native PAGE electrophoresis was performed at 4 °C at 150 V for ~1.5 h. Pol II-containing complexes were detected by scanning FAM fluorescence signal using Typhoon FLA9500 (GE Healthcare) with PMT of 600 (Fig. 5d) or PMT of 700 (Fig. 5e). Three independent experiments were performed (see the source data for Fig. 5).

### Reporting summary

Further information on research design is available in the Nature Portfolio Reporting Summary linked to this article.

## Online content

Any methods, additional references, Nature Portfolio reporting summaries, source data, extended data, supplementary information, acknowledgements, peer review information; details of author contributions and competing interests; and statements of data and code availability are available at 10.1038/s41594-023-01138-w.

### Supplementary information

Reporting Summary

Supplementary Video 1 Morphing of Pol II–SPT6 and Pol II–SPT6–Elongin (structure 1) to show conformational changes upon Elongin binding.

Supplementary Data 1 Protein–protein cross-links identified in Pol II–SPT6–Elongin complexes. Cross-links identified by pLink2.3.11 searches against databases encompassing either only 16 sample-specific proteins and trypsin (DB: ‘16 su + trypsin’) or sample-specific proteins and common MS contaminants (DB: ‘16 su + contaminants’) and filtered at FDR 1 and 5% are shown. The values represent the numbers of CSMs (cross-linked peptide spectrum matches) for each cross-link. ‘Inter-protein’ and ‘Intra-protein’ indicate interprotein and intraprotein cross-links, respectively. ‘Residue 1’ and ‘Residue 2’ are the cross-linked residue pairs in Protein 1 and Protein 2, respectively. ‘UniProt1’ and ‘UniProt2’ stand for UniProtKB accession numbers of Protein 1 and Protein 2, respectively. Note: In this data table, the residue number for ELOA and SPT6 is +3 of the Uniprot numbering/numbering in the structure, because the numbering here starts from the residual residues of the expression tag. Three residues from the expression tag remained at the N-terminus of ELOA and SPT6 after tag cleavage, respectively.

Supplementary Data 2 Primers and templates used for cloning.

### Source data

Source Data Fig. 1 Unprocessed gels for Fig. 1.

Source Data Fig. 1 Statistical source data for Fig. 1.

Source Data Fig. 5 Unprocessed gels for Fig. 5.

Source Data Fig. 5 Statistical source data for Fig. 5.

Source Data Extended Data Fig. 1 Unprocessed gels for Extended Data Fig. 1.

Source Data Extended Data Fig. 1 Statistical source data for Extended Data Fig. 1.

Source Data Extended Data Fig. 2 Unprocessed gels for Extended Data Fig. 2.

Source Data Extended Data Fig. 7 Unprocessed gels for Extended Data Fig. 7.

## Data Availability

The electron density reconstructions and the final four models were deposited into the Electron Microscopy Data Bank (EMDB) and the Protein Data Bank (PDB). The PDB code for the Pol II–SPT6–Elongin complex with ELOA latch (structure 1) is 8OF0, the EMDB code for the local resolution filtered map is EMD-16840 and the EMDB code for the postprocessed map is EMD-16836. The PDB code for the Pol II–Elongin complex lacking the ELOA latch (structure 2) is 8OEW, the EMDB code for the composite map 1 is EMD-16838, and the related focused maps and local resolution filtered maps are EMD-16830, EMD-16831, EMD-16832 and EMD-16839. The PDB code for the Pol II–SPT6–Elongin lacking the ELOA latch (structure 3) is 8OEV, the EMDB code for the composite map 2 is EMD-16837, and the related maps are EMD-16833 and EMD-16834. The PDB code for the Pol II–SPT6 model (structure 4) is 8OEU, and the EMDB code for the composite map 3 is EMD-16835, and related maps are EMD-16828 and EMD-16829. All source files are associated with the manuscript. All mass spectrometry raw files were deposited to the ProteomeXchange Consortium (https://www.proteomexchange.org/) via the PRIDE (Perez-Riverol et al., 2019) partner repository with the dataset identifier PRIDE PXD045446. The PDB codes of previously published structures that were used for structural comparisons are the following: Pol II elongation complex (PDB 5FLM)^28^, Pol II–SPT6–PAF complex (PDB 6GMH)^29^, Pol II-ELL2-EAF1(PDB 7OKX)^36^, human core–PIC in the initial transcribing state without TFIIS present (PDB 5IYD)^37^, human core–PIC in the initial transcribing state with TFIIS present (PDB 5IYC)^37^, paused elongation complex (PEC; PDB 6GML)^38^, PEC–integrator complex (PDB 7PKS)^39^, yeast Pol II at backtracked state (PDB 3PO2)^41^, yeast Pol II–TFIIS complex (PDB 3PO3)^41^, mammalian Pol II–SPT6–PAF–RTF1 complex (PDB 6TED)^27^, mammalian Pol II–SPT6–PAF–RTF1–TFIIS–nucleosome complex (PDB 7UND)^48^, Pol II transcription pre-initiation complex with initial transcription bubble (PDB 7O4I)^43^, the ELOA superfamily homology domain (PDB 4HFX), HIF-1α–pVHL–ELOC–ELOB structure (PDB 1LM8)^34^, Vif–CBFβ–CUL5–ELOB–ELOC complex (PDB 4N9F)^32^, CUL5–RBX2 complex (PDB 6V9I)^31^. Source data are provided with this paper.
