# Supplementary material for: Structure of the transcribing RNA polymerase II–Elongin complex
Source: Nat Struct Mol Biol. 2023 Nov 6;30(12):1925–35. doi: 10.1038/s41594-023-01138-w (PMC10716050; doi:10.1038/s41594-023-01138-w)
Supplement: Supplementary file 1 — Reporting Summary [file 41594_2023_1138_MOESM1_ESM.pdf]

nature portfolio

Corresponding author(s): Patrick Cramer

Last updated by author(s): Sep 20, 2023

Reporting Summary

Nature Portfolio wishes to improve the reproducibility of the work that we publish. This form provides structure for consistency and transparency in reporting. For further information on Nature Portfolio policies, see our Editorial Policies and the Editorial Policy Checklist.

Statistics

For all statistical analyses, confirm that the following items are present in the figure legend, table legend, main text, or Methods section.

n/a	Confirmed	
☐	☒	The exact sample size (*n*) for each experimental group/condition, given as a discrete number and unit of measurement
☐	☒	A statement on whether measurements were taken from distinct samples or whether the same sample was measured repeatedly
☐	☒	The statistical test(s) used AND whether they are one- or two-sided<br>*Only common tests should be described solely by name; describe more complex techniques in the Methods section.*
☒	☐	A description of all covariates tested
☒	☐	A description of any assumptions or corrections, such as tests of normality and adjustment for multiple comparisons
☐	☒	A full description of the statistical parameters including central tendency (e.g. means) or other basic estimates (e.g. regression coefficient) AND variation (e.g. standard deviation) or associated estimates of uncertainty (e.g. confidence intervals)
☐	☒	For null hypothesis testing, the test statistic (e.g. *F*, *t*, *r*) with confidence intervals, effect sizes, degrees of freedom and *P* value noted<br>*Give P values as exact values whenever suitable.*
☒	☐	For Bayesian analysis, information on the choice of priors and Markov chain Monte Carlo settings
☒	☐	For hierarchical and complex designs, identification of the appropriate level for tests and full reporting of outcomes
☒	☐	Estimates of effect sizes (e.g. Cohen's *d*, Pearson's *r*), indicating how they were calculated

Our web collection on statistics for biologists contains articles on many of the points above.

Software and code

Policy information about availability of computer code

Data collection	Orbitrap Exploris 480 4.0.309.28; Xcalibur 4.4.16.14; SerialEM 3.8 beta 8
Data analysis	Relion 3.1; UCSF Chimera 1.13, UCSF ChimeraX v1.11, Pymol 2.3.4, Coot 0.9, Warp v1.0.7-1.0.9, PHENIX 1.18, cryoSPARC 2.14.2, AlphaFold2, Excel, ImageJ, GraphPad Prism 9,pLink (v. 2.3.11)

For manuscripts utilizing custom algorithms or software that are central to the research but not yet described in published literature, software must be made available to editors and reviewers. We strongly encourage code deposition in a community repository (e.g. GitHub). See the Nature Portfolio guidelines for submitting code & software for further information.

Data

Policy information about availability of data

All manuscripts must include a data availability statement. This statement should provide the following information, where applicable:

- Accession codes, unique identifiers, or web links for publicly available datasets
- A description of any restrictions on data availability
- For clinical datasets or third party data, please ensure that the statement adheres to our policy

The electron density reconstructions and the final four models were deposited into the Electron Microscopy Data Base (EMDB) and the Protein Data Bank (PDB). The PDB code for the Pol II-SPT6-Elongin complex with the ELOA latch (structure 1) is 8OF0, the EMDB code for the local resolution filtered map is EMD-16840 and the EMDB code for the postprocessed map is EMD-16836. The PDB code for the Pol II-Elongin complex lacking the ELOA latch (structure 2) is 8OEW, and the EMDB

code for the composite map 1 is EMD-16838, the related focused maps and local resolution filtered maps are: EMD-16830, EMD-16831, EMD-16832 and EMD-16839. The PDB code for the Pol II-SPT6-Elongin lacking the ELOA latch (structure 3) is 8OEV, the EMDB code for the composite map 2 is EMD-16837, and for the related maps are EMD-16833 and EMD-16834. The PDB code for the Pol II-SPT6 model (structure 4) is 8OEU, and the EMDB code for the composite map 3 is EMD-16835, and related maps are EMD-16828, EMD-16829. All source files are associated with the manuscript. All mass spectrometry (MS) raw files were deposited to the ProteomeXchange Consortium (https://www.proteomexchange.org/)via the PRIDE partner repository with the dataset identifier PRIDE: PXD045446.
The PDB codes of previously published structures that were used for structural comparisons are the following: Pol II elongation complex (PDB: 5FLM), Pol II-SPT6-PAF complex (PDB: 6GMH), Pol II-ELL2-EAF1(PDB: 7OKX), human core-PIC in the initial transcribing state without TFIIS present (PDB: 5IYD), human core-PIC in the initial transcribing state without TFIIS present (PDB: 5IYC), paused elongation complex (PEC, PDB: 6GML), PEC-integrator complex (PDB: 7PKS), yeast Pol II at backtracked state(PDB: 3PO2), yeast Pol II-TFIIS complex (PDB: 3PO3), mammalian Pol II-SPT6-PAF-RTF1 complex (PDB: 6TED), mammalian Pol II-SPT6-PAF-RTF1-TFIIS-nucleosome complex (PDB: 7UND), Pol II transcription pre-initiation complex with initial transcription bubble (PDB: 7O4I), the ELOA superfamily homology domain (PDB: 4HFX), HIF-1a-pVHL-ELOC-ELOB structure (PDB: 1LM8), Vif-CBFb-CUL5-ELOB-ELOC complex (PDB: 4N9F), CUL5-RBX2 complex (PDB: 6V9I),the ELOA superfamily homology domain (PDB: 4HFX), HIF-1a-pVHL-ELOC-ELOB structure (PDB: 1LM8), Vif-CBFb-CUL5-ELOB-ELOC complex (PDB: 4N9F), CUL5-RBX2 complex (PDB: 6V9I).

Human research participants

Policy information about studies involving human research participants and Sex and Gender in Research.

Reporting on sex and gender	No human research participants were involved in the study.
Population characteristics	Not applicable
Recruitment	Not applicable
Ethics oversight	Not applicable

Note that full information on the approval of the study protocol must also be provided in the manuscript.

Field-specific reporting

Please select the one below that is the best fit for your research. If you are not sure, read the appropriate sections before making your selection.

☒ Life sciences ☐ Behavioural & social sciences ☐ Ecological, evolutionary & environmental sciences

For a reference copy of the document with all sections, see nature.com/documents/nr-reporting-summary-flat.pdf

Life sciences study design

All studies must disclose on these points even when the disclosure is negative.

Sample size	No statistical methods were used to predetermine sample size. At least three replicates were performed for each assay with quantification. This is according to the common standard for statistical analysis, which requires at least three replicates to calculate means and standard deviations.
Data exclusions	No data were excluded from the analyses.
Replication	All attempts at replication were successful. For statistical data, at lease three independent replicates were performed. Cryo-EM single particle analysis inherently relies on averaging over a large number of independent observations.
Randomization	Samples were not allocated to groups.
Blinding	Investigators were not blinded during data acquisition and analysis because it is not a common procedure for the methods employed. Blinding was not employed for three reasons. 1)This study is not a confirmatory research and it can be repeated easily. 2) Unlike clinical research, where blinding is a common procedure, this study did not involve human subjects, and bias due to psychological effect of the subjects is not a risk. 3) Proper controls are included in the study to make sure reliable conclusions and avoid biases from the experimenter.

Reporting for specific materials, systems and methods

We require information from authors about some types of materials, experimental systems and methods used in many studies. Here, indicate whether each material, system or method listed is relevant to your study. If you are not sure if a list item applies to your research, read the appropriate section before selecting a response.

Materials & experimental systems

n/a	Involved in the study
☒	☐ Antibodies
☐	☒ Eukaryotic cell lines
☒	☐ Palaeontology and archaeology
☒	☐ Animals and other organisms
☒	☐ Clinical data
☒	☐ Dual use research of concern

Methods

n/a	Involved in the study
☒	☐ ChIP-seq
☒	☐ Flow cytometry
☒	☐ MRI-based neuroimaging

Eukaryotic cell lines

Policy information about cell lines and Sex and Gender in Research

Cell line source(s)	Hi5 cells: Expression Systems, Trichoplusia ni Insect cells in ESF921 media, item 94-002F<br>Sf9 cells: ThermoFisher, Catalogue Number 12659017, Sf9 cells in Sf-9000TM III SFM Sf21 cells: Expression Systems, SF21 insect cells in ESF921 medium, Item 94-003F
Authentication	None of the cell lines were authenticated.
Mycoplasma contamination	Cell lines were not tested for mycoplasma contamination.
Commonly misidentified lines (See ICLAC register)	No commonly misidentified cell lines were used.
