# Supplementary material for: Structure of the transcribing RNA polymerase II–Elongin complex
Source: Nat Struct Mol Biol. 2023 Nov 6;30(12):1925–35. doi: 10.1038/s41594-023-01138-w (PMC10716050; doi:10.1038/s41594-023-01138-w)

Source data for Fig. 1

Original gels, 50% scaling, no other changes, regions in use are boxed

1a

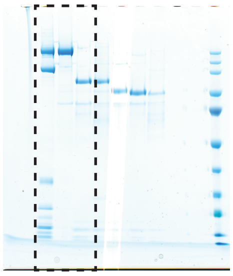

1b   22.04.2022 Replicate 1

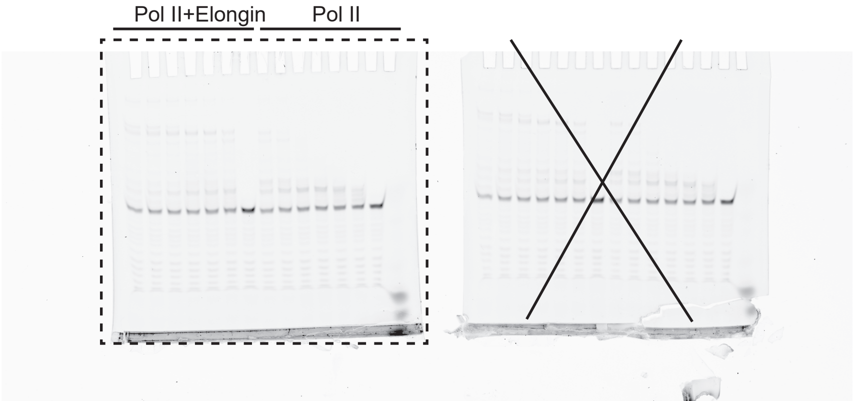

19.05.2022 Replicate 2

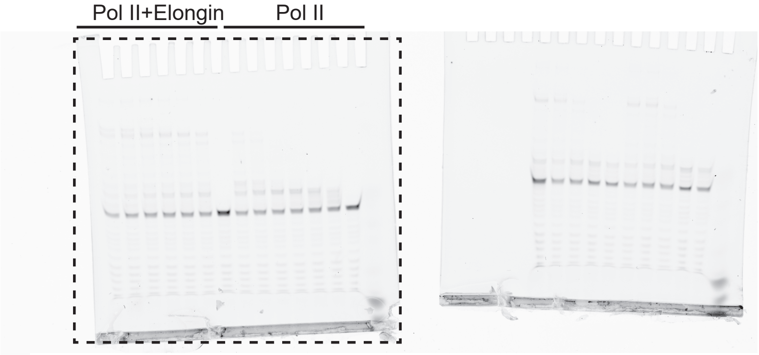

23.05.2022 Replicate 3, showed in Fig.1b as the representative gel

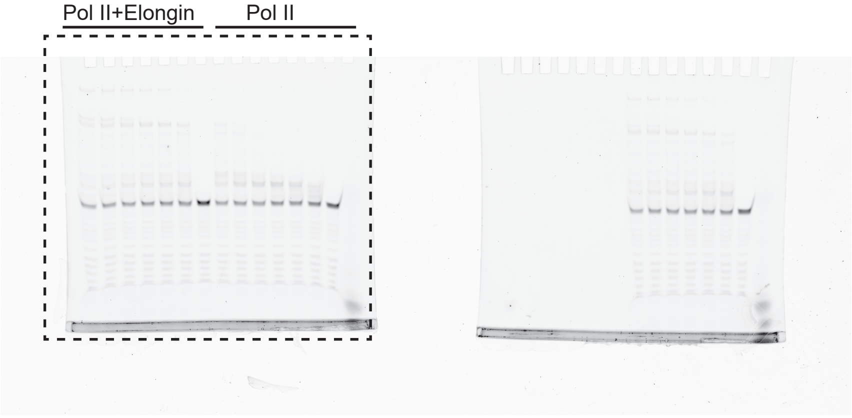

Supplement: Supplementary file 5 — Unprocessed gels for Fig. 1. [file 41594_2023_1138_MOESM5_ESM.pdf]
