# Supplementary material for: Structure of the transcribing RNA polymerase II–Elongin complex
Source: Nat Struct Mol Biol. 2023 Nov 6;30(12):1925–35. doi: 10.1038/s41594-023-01138-w (PMC10716050; doi:10.1038/s41594-023-01138-w)

Source Data for Fig. 5 & Extended Data Fig. 7

Original gels, 50% scaling, no other changes, regions in use are boxed

Fig. 5b & Extended Data Fig. 7a

Replicate 1, 23.06.2022

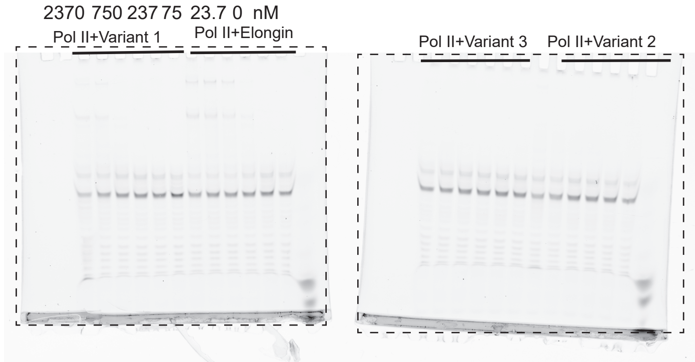

Replicate 2, 24.06.2022

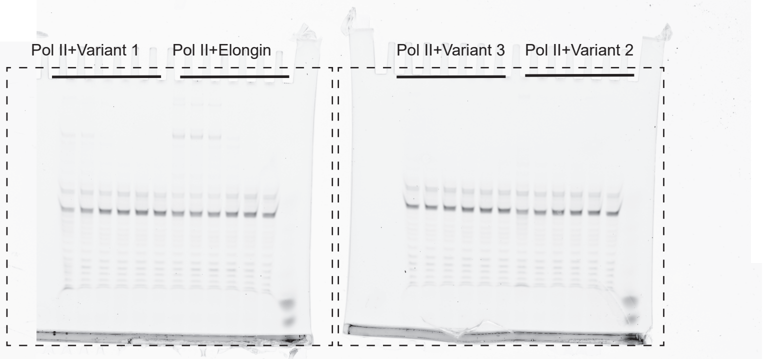

Replicate 3, 30.06.2022

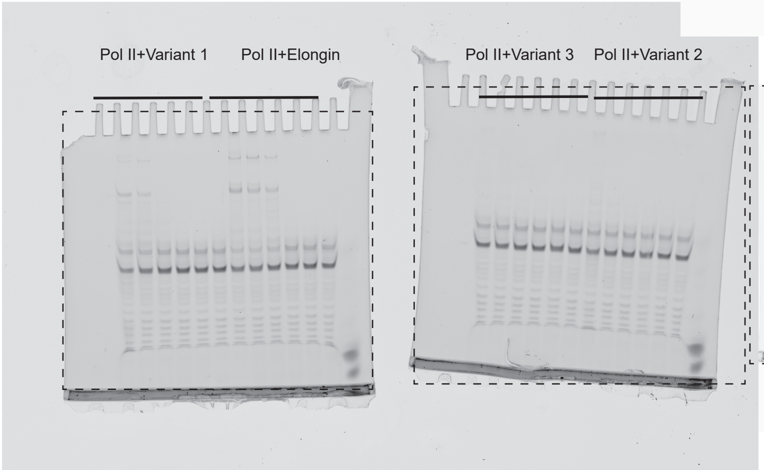

Replicate 4, 01.07.2022

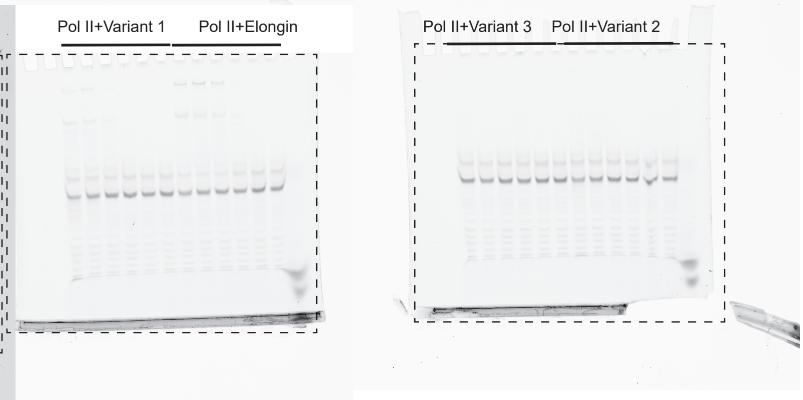

Fig. 5c & Extended Data Fig. 7b

23.12.2022 Replicate 1

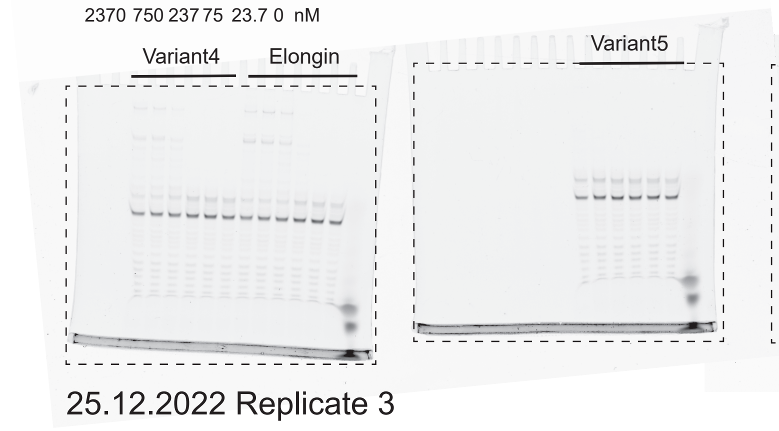

24.12.2022 Replicate 2

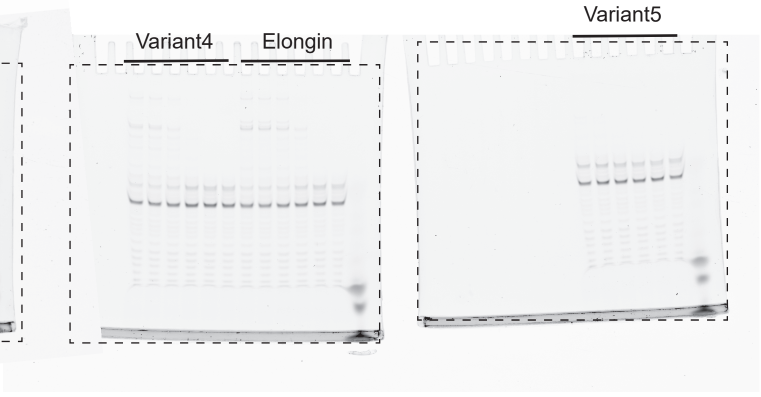

25.12.2022 Replicate 3

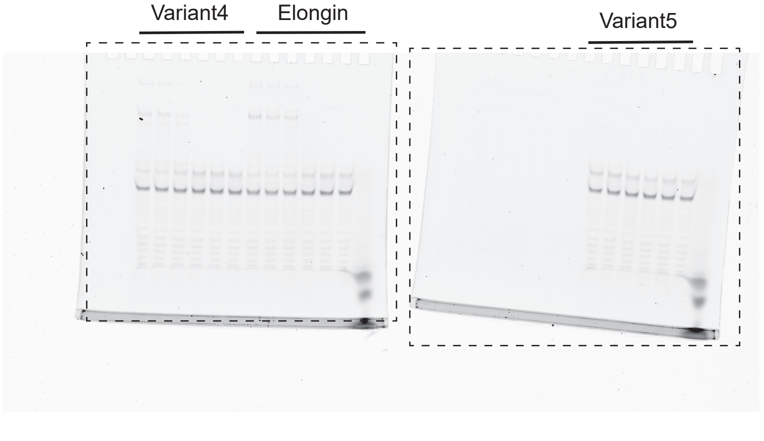

Source Data for Fig. 5d

Original gels, no scaling, no other changes, regions in use are boxed

d Replicate 1, 17.08.2022

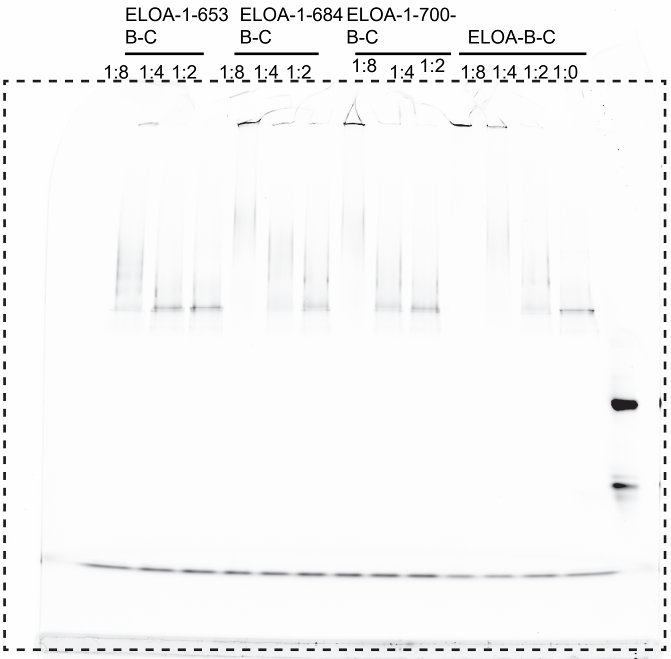

d Replicate 2, 18.08.2022

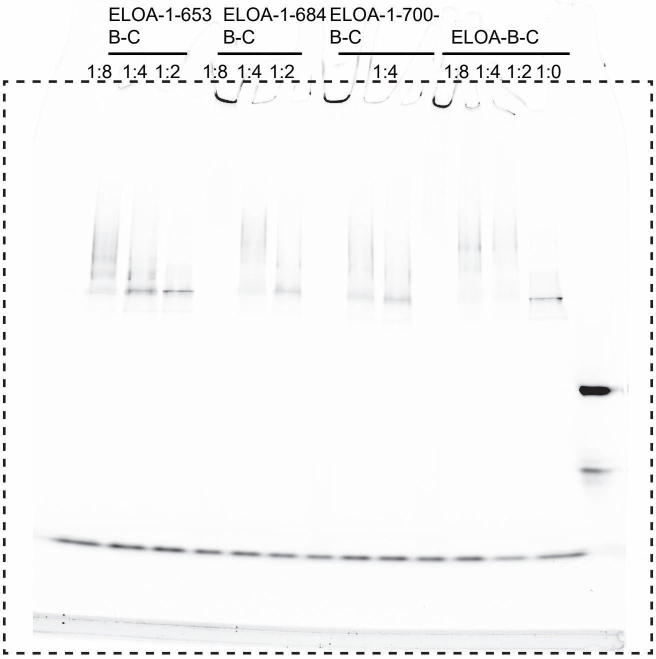

d Replicate 3, 19.08.2022

shown as representative gel in the figure

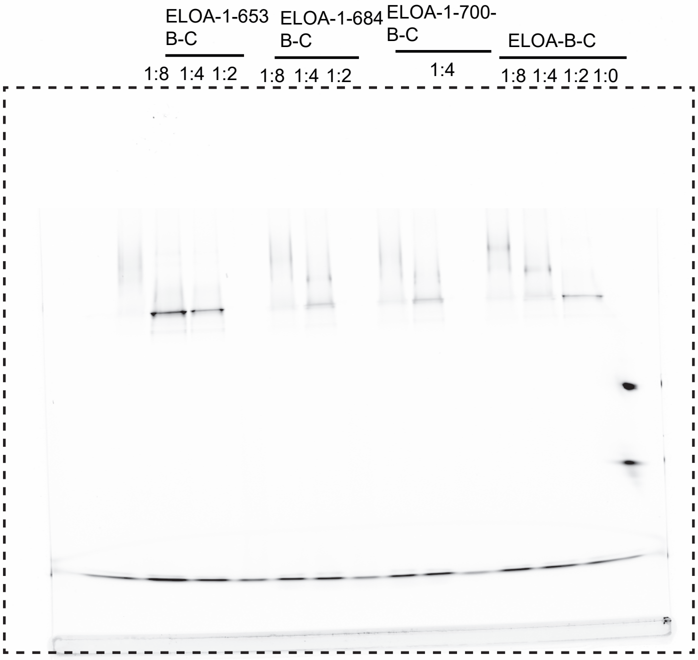

Source Data for Fig. 5e

Original gels, no scaling, no other changes, regions in use are boxed

e Replicate 1, 23.12.2022

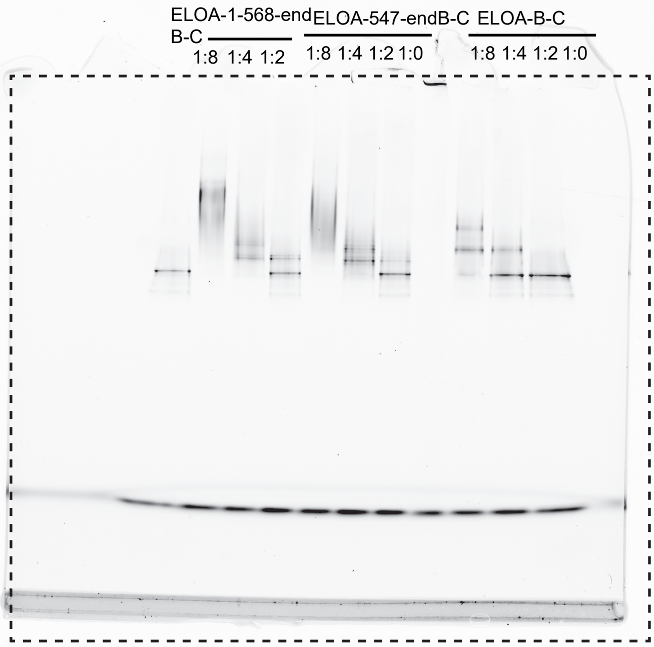

e Replicate 2, 24.12.2022

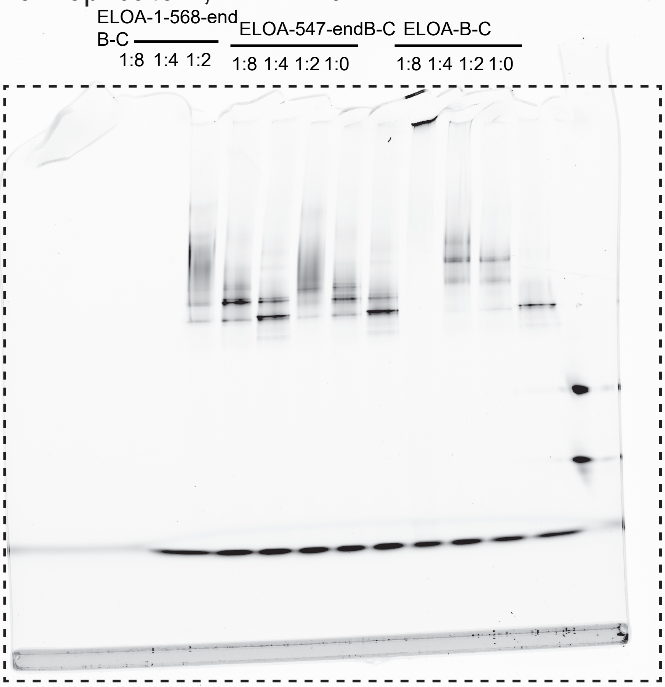

shown as representative gel in the figure

e Replicate 3, 25.12.2022

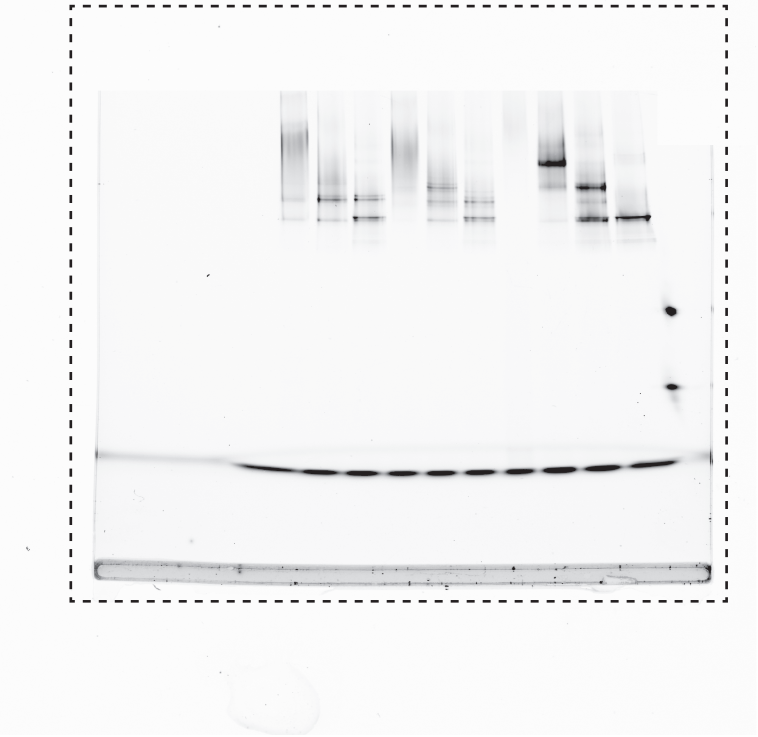

Supplement: Supplementary file 7 — Unprocessed gels for Fig. 5. [file 41594_2023_1138_MOESM7_ESM.pdf]
