# Supplementary material for: Structure of the transcribing RNA polymerase II–Elongin complex
Source: Nat Struct Mol Biol. 2023 Nov 6;30(12):1925–35. doi: 10.1038/s41594-023-01138-w (PMC10716050; doi:10.1038/s41594-023-01138-w)

Source Data_Extended Data Fig. 1
Original gels, 50% scaling, no other changes, regions in use are boxed

1B-1C

22.04.2022 Replicate 1

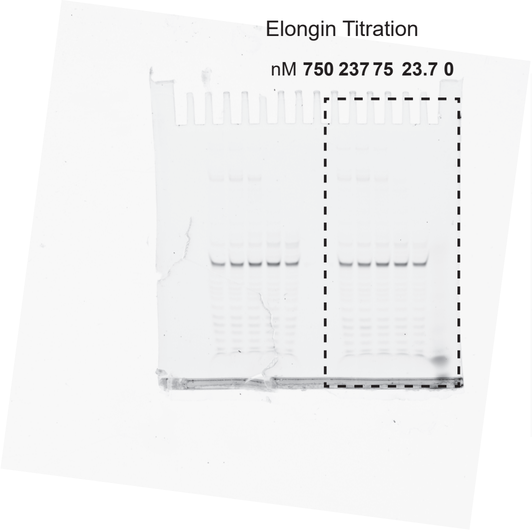

19.05.2022 Replicate 2 and 3

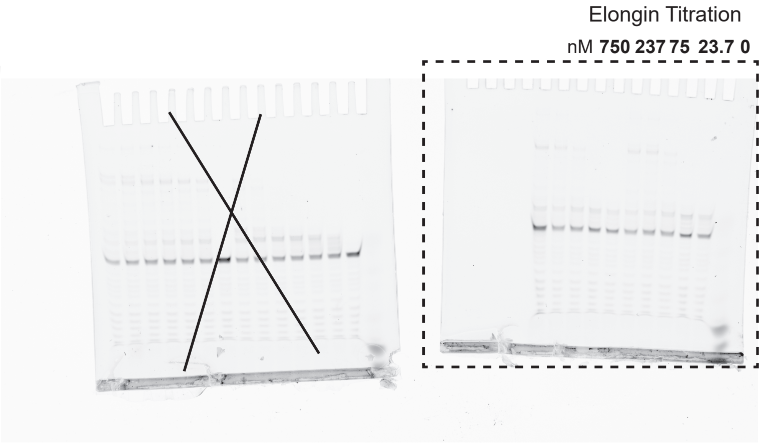

Supplement: Supplementary file 9 — Unprocessed gels for Extended Data Fig. 1. [file 41594_2023_1138_MOESM9_ESM.pdf]
