# Supplementary material for: Structure of the transcribing RNA polymerase II–Elongin complex
Source: Nat Struct Mol Biol. 2023 Nov 6;30(12):1925–35. doi: 10.1038/s41594-023-01138-w (PMC10716050; doi:10.1038/s41594-023-01138-w)

Source data for Extended Data Fig. 2

Original gels, 50% scaling, no other changes, regions in use are boxed

2c

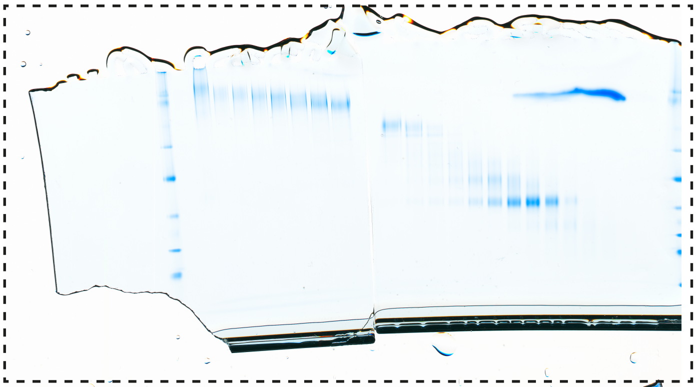

2b

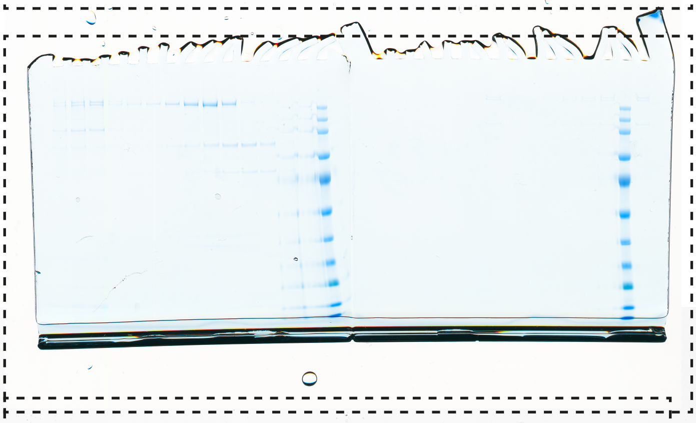

2c

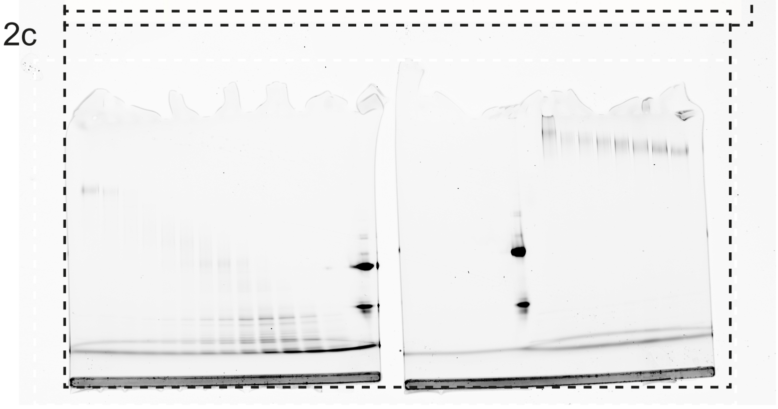

Supplement: Supplementary file 11 — Unprocessed gels for Extended Data Fig. 2. [file 41594_2023_1138_MOESM11_ESM.pdf]
