# Supplementary figures and images for: Structure of the transcribing RNA polymerase II–Elongin complex
Source: Nat Struct Mol Biol. 2023 Nov 6;30(12):1925–35. doi: 10.1038/s41594-023-01138-w (PMC10716050; doi:10.1038/s41594-023-01138-w)

Source data for Extended Data Fig. 7 c,d

Original gels, regions in use are boxed

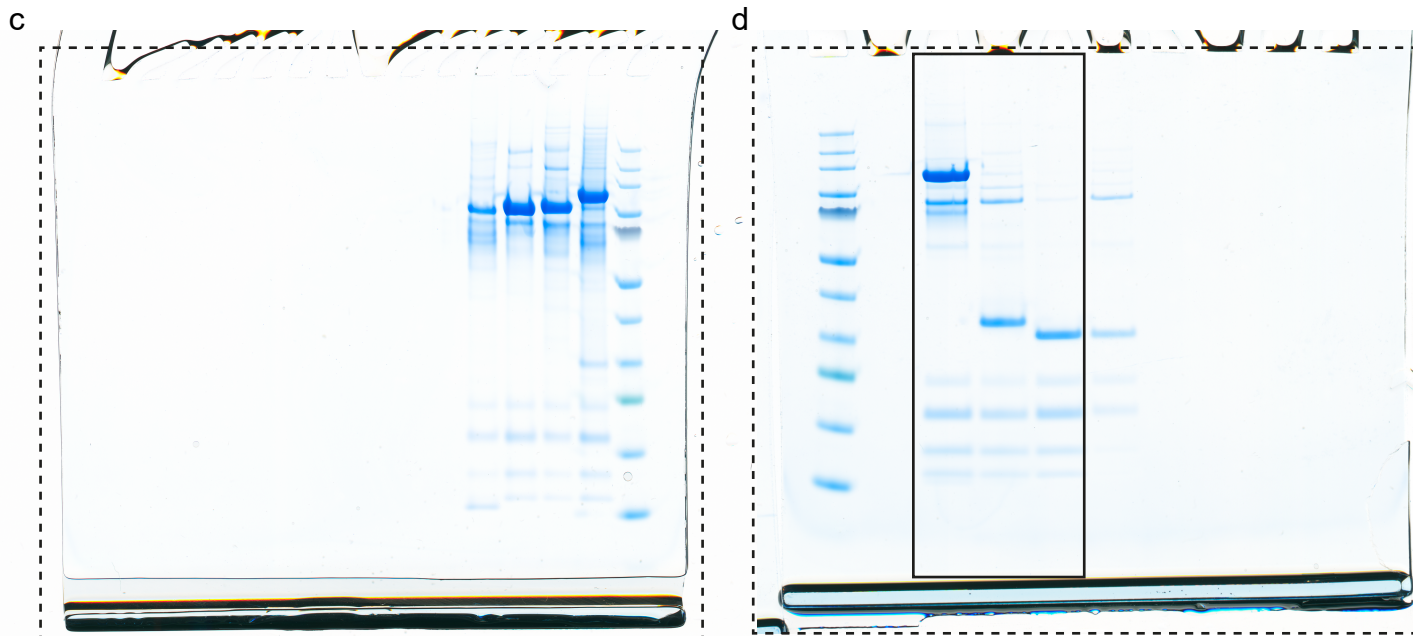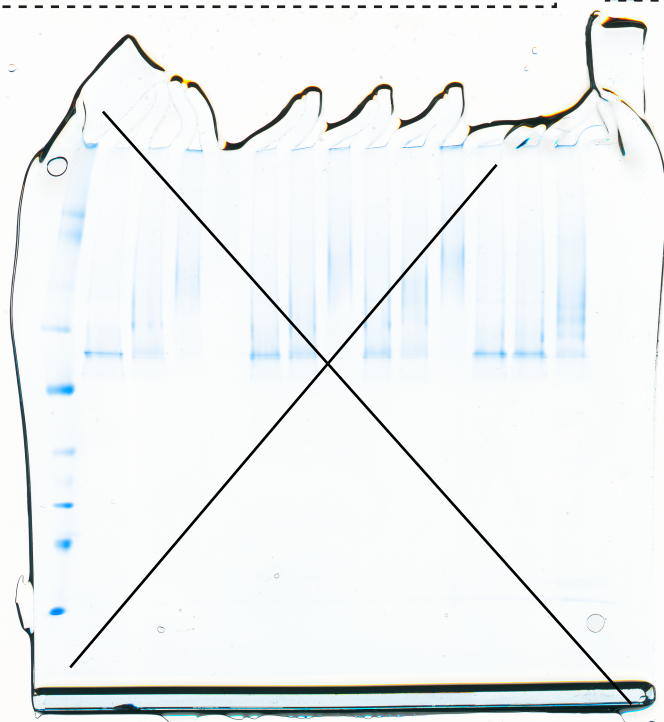

Supplement: Supplementary file 12 — Unprocessed gels for Extended Data Fig. 7. [file 41594_2023_1138_MOESM12_ESM.pdf]
